# A central role for TRPS1 in the control of cell cycle and cancer development

**DOI:** 10.18632/oncotarget.2291

**Published:** 2014-07-31

**Authors:** Lele Wu, Yuzhi Wang, Yan Liu, Shiyi Yu, Hao Xie, Xingjuan Shi, Sheng Qin, Fei Ma, Tuan Zea Tan, Jean Paul Thiery, Liming Chen

**Affiliations:** ^1^ The Key Laboratory of Developmental Genes and Human Disease, Ministry of Education, Institute of Life Science, Southeast University, Nanjing, PR China; ^2^ Laboratory for Comparative Genomics and Bioinformatics & Jiangsu Key Laboratory for Biodiversity and Biotechnology, College of Life Science, Nanjing Normal University, Nanjing, China; ^3^ Cancer Science Institute, National University of Singapore, 14 Medical Drive, Singapore; ^4^ Institute of Molecular and Cell Biology, A*STAR, 61 Biopolis Drive, Singapore; ^5^ Department of Biochemistry, Yong Loo Lin School of Medicine, National University of Singapore, 8 Medical Drive, Singapore

**Keywords:** Cancer development, Cell cycle control, HDAC, Histone acetylation, TRPS1

## Abstract

The eukaryotic cell cycle is controlled by a complex regulatory network, which is still poorly understood. Here we demonstrate that TRPS1, an atypical GATA factor, modulates cell proliferation and controls cell cycle progression. Silencing TRPS1 had a differential effect on the expression of nine key cell cycle-related genes. Eight of these genes are known to be involved in the regulation of the G2 phase and the G2/M transition of the cell cycle. Using cell synchronization studies, we confirmed that TRPS1 plays an important role in the control of cells in these phases of the cell cycle. We also show that silencing TRPS1 controls the expression of 53BP1, but not TP53. TRPS1 silencing also decreases the expression of two histone deacetylases, HDAC2 and HDAC4, as well as the overall HDAC activity in the cells, and leads to the subsequent increase in the acetylation of histone4 K16 but not of histone3 K9 or K18. Finally, we demonstrate that TRPS1 expression is elevated in luminal breast cancer cells and luminal breast cancer tissues as compared with other breast cancer subtypes. Overall, our study proposes that TRPS1 acts as a central hub in the control of cell cycle and proliferation during cancer development.

## INTRODUCTION

Cells proliferate rapidly during embryonic and postnatal development,[1] and studies show that deregulation of proliferation and a reduction in the degree of apoptosis are necessary steps in cancer initiation and progression.[2] There are various mechanisms driving cell cycle progression, which are central to understanding how cancer is initiated.[3] Indeed, numerous cell cycle-related genes, including cyclins, cyclin-dependent kinases and histone modification enzymes such as histone deacetylases (HDACs), have been characterized to play critical roles in cell cycle progression.[4, 5] However, the networks regulating cell cycle progression are still poorly understood.

*Trps1*, mapped to human chromosome 8q23-24, is implicated in trichorhinophalangeal syndrome (TRPS),[6-11] also known as Langer-Giedion syndrome, a genetic disorder characterized by short stature, cone-shaped ends of the long bones (epiphyses), and distinctive facial features linked to skeletal abnormalities. TRPS1 is an atypical GATA protein containing three distinct zinc finger domains, C2H2, GATA, and Ikaros, which bind to GATA sequences and regulate gene expression by repressing the transcriptional activation of other GATA factors. Transcriptional repression by TRPS1 depends on the modulation of its C-terminal repressor region (RG) through SUMOylation[12]—rather than through competition for DNA binding[13, 14]—and studies show that TRPS1 is directly inhibited by the dynein light chain 8 protein (LC8a) and RING finger protein 4 (RNF4).[15] Besides repressing transcription, TRPS1 was contrarily found to activate the transcription of Wnt inhibitors, such as *Wif1*, *Apcdd1* and *Dkk4* in the developing vibrissa follicle, by directly binding to their promoters.[16]

TRPS1 function has been predominantly elucidated in bone, hair follicles and kidney during the development and differentiation of these structures. During chondrocyte proliferation and differentiation, TRPS1 has been shown to repress the expression of PTHrP[17] and osteocalcin[18] via direct interaction with their promoters, and physically interacts with Runx2 to prevent Runx2-mediated trans-activation.[19] TRPS1 also suppresses the expression of GLI3[20] by interacting with its transactivation domain. Studies also indicate that TRPS1 interacts with and increases the activities of HDAC1 and HDAC4 to reduce histone H3K9 and K18 acetylation during mitosis.[21] TRPS1 also promotes chondrocytic proliferation and apoptosis by repressing the expression of *Stat3*,[22] and acts downstream of GDF5 to promote the differentiation and apoptosis of ATDC5 chondrogenic cells.[23] In hair follicles, TRPS1 represses *Runx1* expression via binding to the GATA domain of the P2 promoter of *Runx1*,[24] and also represses the expression of the hair follicle stem cell regulator Sox9 to control proliferation of the follicle epithelium.[25] In the kidney, TRPS1 is induced by BMP7 to modulate the BMP7-induced mesenchymal-to-epithelial (MET) transition during development,[26] and represses the activation of TGFβ/SMAD3 signaling pathway to promote ureteric bud branching.[27] Furthermore, *Trps1* haploinsufficiency has been linked to renal fibrosis, which is thought to manifest through an increase in SMAD3 phosphorylation and E3-ubiquitin ligase Arkadia expression, concomitant with a decrease in SMAD7 to promote TGFβ1-mediated epithelial-to-mesenchymal transition (EMT).[28] However, the potential role of TRPS1 in cell proliferation or in the control of the cell cycle in bone, in the hair follicle or in the kidney is largely unknown.

In addition to its role in development, TRPS1 has been implicated in human cancers, including prostate cancer,[13, 29, 30] leukemia,[31] colon cancer,[32] endometrial cancer,[33] and breast cancer.[34-40] As a critical regulator of MET and EMT in cancer,[36, 41-43] TRPS1 is reported to negatively regulate ZEB2 in EMT and its knockdown causes a decrease in *E-cadherin* mRNA but an increase in *Vimentin* mRNA in breast cancer.[41] More recent work demonstrates that microRNA-221/222 targets TRPS1 to induce EMT in breast cancer[43] and that TRPS1 down-regulation by miRNA-221 is essential for platelet-derived growth factor (PDGF)-mediated EMT in pancreatic cancer cells.[44] Studies have yet to confirm a role for TRPS1 in cell proliferation or cell cycle control as it pertains to cancer.

In this study, we sought to ascertain a role for TRPS1 in cellular proliferation and cell cycle in cancer cell lines and tumor samples. We found that TRPS1 modulates cell proliferation by controlling the cell cycle but has no role in the regulation of apoptosis. We show that TRPS1 affects the expression of nine key cell cycle genes, and confirm the regulatory role of TRPS1 during the G2-phase and the G2/M transition of the cell cycle. Furthermore, we demonstrate that TRPS1 silencing decreases HDAC activity, which in turn leads to an increase in histone4 K16 acetylation. TRPS1 was also shown to control the expression of 53BP1 but not TP53. Finally, we show a higher expression of TRPS1 in luminal breast cancer cells and luminal breast cancer patient samples as compared with basal breast cancer cells and basal breast cancers patient samples, respectively. Taken together, our findings have deciphered a central role for TPRS1 in the regulatory network controlling the cell cycle and cancer development.

## RESULTS

### TRPS1 modulates cancer cell proliferation through cell cycle regulation

**Figure 1:**
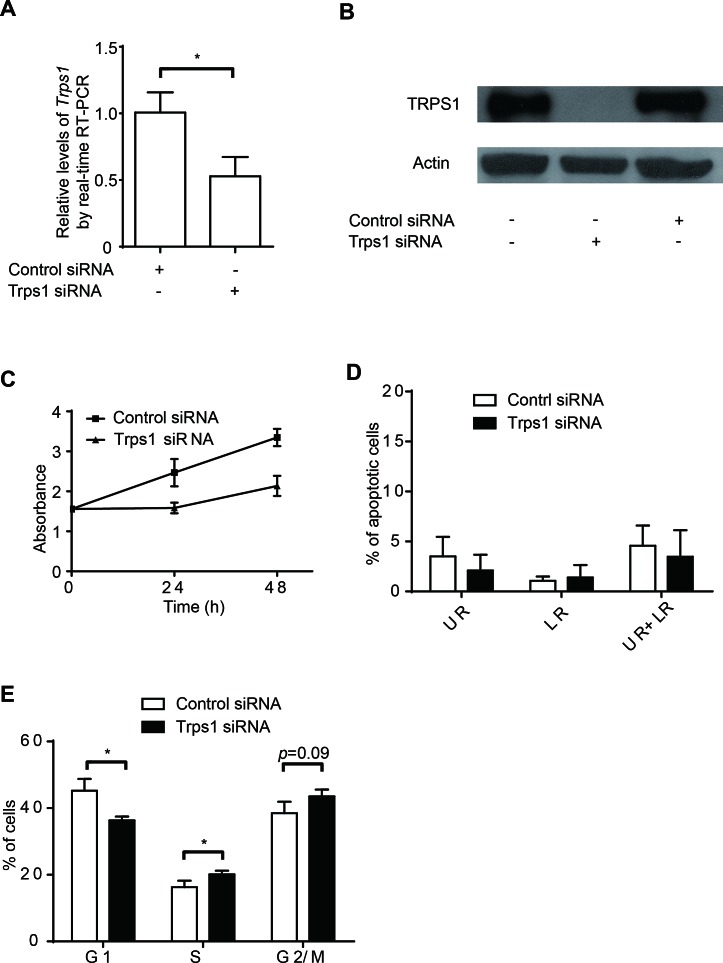
TRPS1 promotes cell proliferation by controlling cell cycle progression (A-B) TRPS1 was significantly silenced by siRNA at both the (A) mRNA and (B) protein levels; (C-D) Knockdown of TRPS1 caused a (C) decrease in cell proliferation but it had no effect on apoptosis (D). (E) Cell cycle progression was affected following TRPS1 silencing. *p <0.05.

Given the relative paucity of information concerning TRPS1 during cell proliferation as compared with its role in other aspects of cancer, we first sought to assess the role of this transcriptional repressor in cell proliferation and cell cycle using an siRNA approach. Using BT474 human breast cancer cells, we first confirmed that TRPS1 could be successfully knocked down by siRNA at both the mRNA and protein levels (Figure 1A and B). A total elimination of TRPS1 protein with 50% reduction at Trps1 mRNA using siRNA pool against Trps1 indicates that the siRNA pool against Trps1 was able to repress gene expression via both inhibiting Trps1 translation and degrading Trps1 mRNA. This knockdown of TRPS1 led to a significant decrease in BT474 cell proliferation (Figure 1C). Although TRPS1 was previously linked to apoptosis of prostate cancer cells[13, 29, 45] and chondrocytes,[22] we found that TRPS1 silencing had little effect on BT474 cell apoptosis (Figure 1D). Thus, we hypothesized that TRPS1 may contribute to cell cycle regulation to promote BT474 cell proliferation. Indeed, following TRPS1 knockdown, we found that BT474 cells accumulated in the S-phase and G2/M transition phase (Figure 1E). Thus, TRPS1 appears to modulate BT474 cell proliferation by controlling the cell cycle without affecting BT474 cell apoptosis.

### TRPS1 controls the expression of several key cell cycle genes

**Figure 2:**
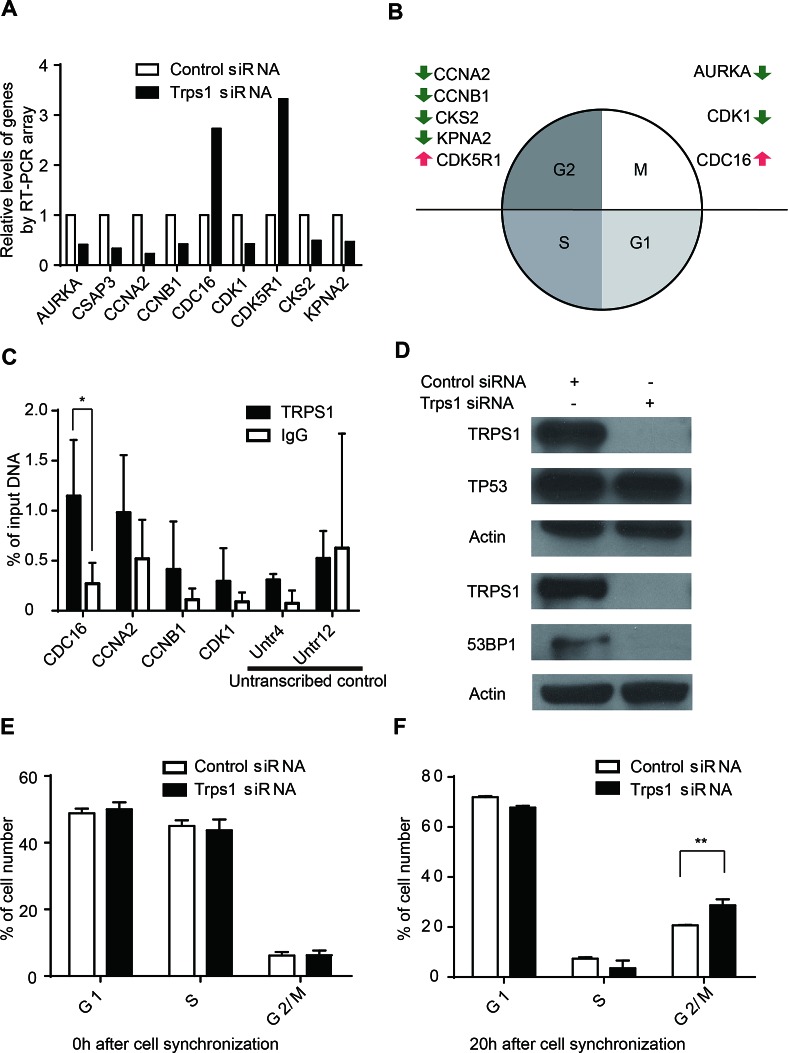
TRPS1 regulates cell cycle progression by controlling the expression of cycle cell genes (A) Significantly changed cell cycle-related genes (>2-fold or <0.5-fold) after TRPS1 silencing. (B) Functional annotation of cell cycle-related genes with respect to the cell cycle. Green and red arrows represent down- and up-regulated gene expression after TRPS1 knockdown. (C) DNA binding activity of TRPS1 on cell cycle gene promoters determined by ChIP-qPCR analysis. *p <0.05. (D) Western blotting showed that 53BP1 was down-regulated whereas TP53 was unaffected after TRPS1 knockdown. (E) Cells were successfully synchronized to G1/S stage using a double thymidine block (0 h). (F) There was a significant accumulation of *Trps1*-knockdown cells in the G2/M phase at 20 h after release from synchronization. **p <0.005.

To identify which cell cycle genes might be affected by TRPS1, we used an RT-PCR cell cycle array of 84 cell cycle-related genes. We identified nine key cell cycle genes significantly regulated by TRPS1 (Figure 2A and Figure S1), wh­ich are predominantly involved in the regulation of the G2 phase and the G2/M transition of the cell cycle (Figure 2B). In these nine genes, only CDC16 and CCNA2 contain GATA binding motif- W(A/T)GATAR(A/G). We found that TRPS1 enriched on the promoters of CDC16 and CCNA2 compared to CCNB1 and CDK1 containing GATA but not W(A/T)GATAR(A/G) motifs in their promoters (Figure 2C). These results indicate that CDC16 and CCNA2 might be direct targets of TRPS1, while other cell genes might be indirectly regulated by TRPS1. Upon TRPS1 silencing, AKT, p-AKT, ERK1/2, p-ERK1/2 and GSK3β didn't show significant changes (Figure S2). These results indicated that TRPS1 function might not involve EGF-R/HER2 signaling pathway in BT474 cell line. Next we examined TP53 status, because more than half of all human cancers, including breast cancers, lose TP53 function[46] and TP53 is a known master regulator of cell-cycle checkpoints.[47] However, TRPS1 silencing had no effect on TP53 expression, as determined using RT-PCR cell cycle arrays. We further validated this finding, showing that the TP53 protein level was also unaffected after TRPS1 knockdown using western blotting (Figure 2D). A previous study indicated that TRPS1 may be related to 53BP1, although the biological effects of this TRPS1-53BP1 interaction remain uncharacterized.[48] The 53BP1 protein also functions as an inhibitor of BRCA1 accumulation at double stranded break sites in the G1 phase of the cell cycle.[49] Interestingly, we found that TRPS1 knockdown downregulated the expression of 53BP1 (Figure 2D).

In order to further verify whether TRPS1 was involved in G2/M control, a double thymidine block was used to synchronize BT474 cells to the G1/S phase (Figure 2E). After release of the block, we observed a significant accumulation of BT474 cells in the G2/M phase (Figure 2F). Taken together, our results indicate that TRPS1 has a regulatory role on the cell cycle by affecting the expression of key cell cycle genes important for G2 phase and G2/M transition of the cell cycle.

### TRPS1 regulates HDAC activity and controls the expression HDAC2 and -4 to modulate histone acetylation

**Figure 3:**
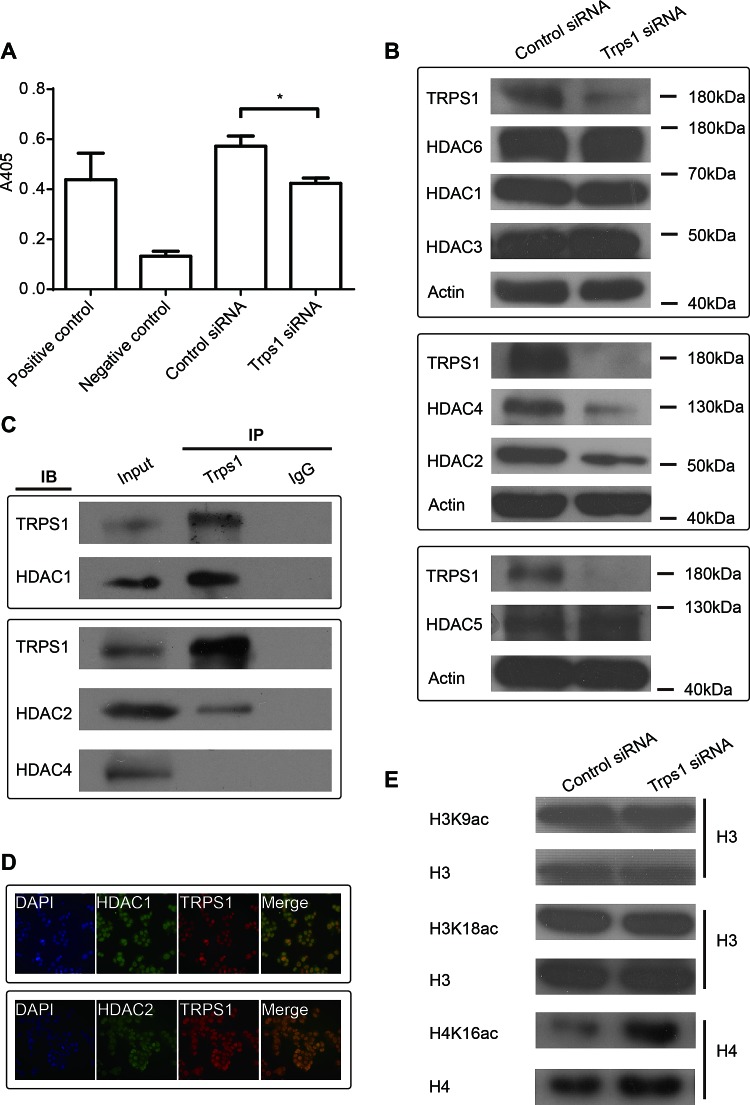
TRPS1 controls the activity and expression of HDAC as well as histone acetylation (A) HDAC activity was decreased after TRPS1 knockdown. Absorbance was measured at 405 nm (A405, y-axis; *p<0.05). (B) HDAC2, HDAC4 and TRPS1 protein expressions were decreased after TRPS1 silencing. Actin was used as a loading control in each blot. (C) HDAC1 and -2 not HDAC4 co-immunoprecipitated with TRPS1. (D) HDAC1 and -2 coloclaized with TRPS1. (E) Histone 4 K16 acetylation was increased after TRPS1 knockdown.

TRPS1 was reported to be able to modulate HDAC activity to control histone3 K9 and K18 acetylation during mitosis in chondrocytes.[21] We investigated whether TRPS1 also modulates HDAC activity in cancer cells and found that HDAC activity was decreased following TRPS1 knockdown (Figure 3A). Furthermore, TRPS1 knockdown led to a decrease in the expression of HDAC2 and -4 but not HDAC1, -3, -5 or -6 (Figure 3B), suggesting that TRPS1 modulates HDAC activity by controlling the expression of specific HDACs. The proteins immunoprecipitated by TRPS1 antibodies and control IgG were subjected to MS analysis to reveal the TRPS1 interactomes (Supplementary Table 1). HDAC1 and HDAC2 not other HDACs were identified as the TRPS1 interacting candidates. We further validated that TRPS1 interacted HDAC1 and -2 but not HDAC4 using CO-IP (Figure 3D) and TRPS1 was found to be colocalized with HDAC1 and -2 (Figure 3E). Previous findings in chondrocytes show that TRPS1 negatively regulates the acetylation of H3K9 and H3K18.[21] Thus, we next tested the effect of TRPS1 on their acetylation in cancer cells. However, we found that H3K9 and H3K18 acetylation was not affected by TRPS1 knockdown in these cells but that H4K16 acetylation was increased following TRPS1 silencing (Figure 3C). On the one hand, these results confirm the complexity and specificity of different members of the HDAC family in their regulation of histone modification; on the other hand, these findings indicate that the epigenetic consequences of TRPS1 regulation of HDACs are cell type- and context-dependent. Nevertheless, these results suggest that TRPS1 regulates HDAC activity to modulate histone4 K16 acetylation, and this process presumably involves TRPS1 control over the expression of certain HDAC members.

### TRPS1 expression is elevated in luminal breast cancer cells and luminal breast cancers

**Figure 4:**
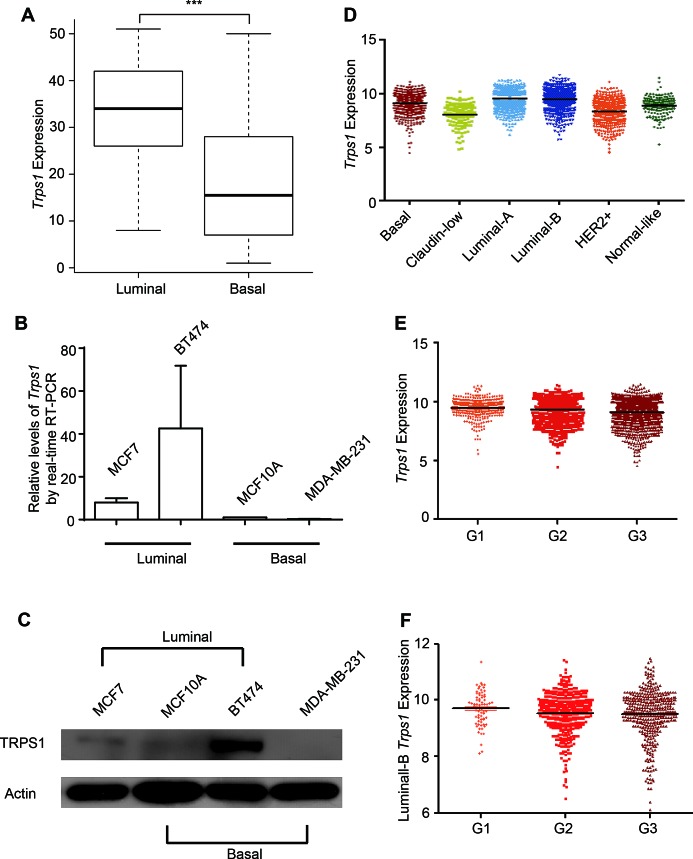
TRPS1 in breast cancer cell lines and breast cancers (A) *Trps1* was elevated in Luminal breast cancer cell lines as compared with Basal breast cancer cell lines using the expression data of 51 breast cancer cell lines.[50] ***p=2.7774e-04. (B-C) MCF7, BT474, MCF10A and MDA-MB-231 cell lines were used to validate TRPS1 expression at the (B) mRNA and (C) protein levels. (D) *Trps1* expression in different breast cancer subtypes from patient samples. Basal subtype versus (*vs*) the remaining samples (Rest), *p*=6.48e-07; Claudin-Low *vs* Rest, *p*=2.66e-33; Luminal-A *vs* Rest, *p*=9.66e-46; Luminal-B *vs* Rest, *p*=1.69e-35; ERBB2+ *vs* Rest, *p*=2.66e-95; Normal-Like *vs* Rest, *p*=7.50e-13. (E) *Trps1* expression in different breast cancer stages. Grade (G) 1 *vs* G2, *p*=2.27e-03; G1 *vs* G3, *p*=8.65e-14; G2 *vs* G3, *p*=1.12e-09. (F) *Trps1* expression in Luminal-B breast cancer stages. G1 *vs* G2, *p*=0.0826; G1 *vs* G3, *p*=0.08; G2 *vs* G3, *p*=0.8159.

TRPS1 was previously identified to be overexpressed in breast cancer,[38] and higher TRPS1 expression is significantly associated with estrogen receptor (ER)-α, progesterone receptor (PR), and human epidermal growth factor receptor 2 (HER2) expression, as well as improved survival in breast cancer patients.[37] Consistently, we found that TRPS1 expression was significantly elevated in luminal breast cancer cells as compared with basal breast cancer cells using the expression data of 51 breast cancer cell lines[50] (Figure 4A). This was further validated by the elevated TRPS1 expression in two luminal breast cancer cell lines (MCF7 and BT474 cells) as compared with the basal breast cancer cell line, MDA-MB-231, and non-cancerous MCF10A cells, which are classified as basal subtype according to Neve's published work [50] (Figure 4B and C). Among the investigated breast cancer cell lines, TRPS1 expression was highest in BT474 cells and lesser in MCF7 cells, whereas it was almost undetectable in MDA-MB-231 cells (Figure 4B and C). In primary breast tumor samples, elevated expression of *Trps1* was observed in luminal-A and -B breast cancers (Figure 4D). However, we found a lower *Trps1* expression in HER2+ breast cancers as compared with basal breast cancer samples (Figure 4D). TRPS1 expression also systematically decreased from Grade 1 to Grade 3 breast cancer across the entire cohort (Figure 4E), as well as in luminal-B breast cancers when examined separately (Figure 4F). Overall, our findings are in line with previous results,[34, 37] showing that TRPS1 has both an oncogenic and a tumor suppressor role in breast cancer development.

## DISCUSSION

Normal tissue architecture and function is maintained through strict limitations on cell number, which is achieved via stringent cell cycle regulation.[51] Indeed, the mechanism(s) governing cell cycle transitions is central in the study of cell proliferation[3] and it is presumed that aberrations to this cell cycle control are partly responsible for the onset and development of tumor. Here, we sought to ascertain a role for TRPS1 in cell cycle control and cell proliferation in cancer cells and tissue samples. We found that TRPS1 mediates BT474 cell proliferation via regulating cell cycle progression, and has no effect on apoptosis. Following TRPS1 knockdown, we found that BT474 cells accumulate in the S and G2/M stages, and that 9 of 84 cell cycle-related genes are up- or down-regulated. We also showed that 53BP1 but not TP53 is modulated by TRPS1. These findings together indicate that TRPS1 regulates cell cycle transition by modulating the expression of key genes involved in cell cycle progression.

Of the nine genes regulated by TRPS1, eight (CDK1, CCNA2, CCNB1, CKS2, KPNA2, AURKA, CDK5R1 and CDC16) have been implicated during the G2-phase and G2/M transition. Cdk1 is thought to be the major kinase that initiates the onset of mitosis, whereas cyclins A2 (CCNA2) and B1 control the G2/M transition.[52] CCNA2 is synthesized in somatic cells at the onset of DNA synthesis and is associated with cyclin-dependent protein kinase 1 (CDK1) and CDK2 during G2/M transition.[53] CCNB1, on the other hand, is cell cycle regulated and peaks during the G2/M transition.[54] Impaired transcription of *CCNB1*, *CCNA2* and *CDK1* contributes to mouse embryonic fibroblast cell cycle arrest in G2.[55] Cdks are also found in complex with another of the altered genes, CDK5R1, to create the E2F1/Cdk/CDK5R1 complex, which forms in the nucleus to prevent the advancement of the cell cycle.[56] Loss of function of Cdk-associated proteins (Cks) leads to mitotic defects in yeast *Saccharomyces cerevisiae*.[57] Indeed, it has been shown that silencing of Cks1 and Cks2, the two paralogs of yeast Cks, in mouse embryonic fibroblasts leads to cell cycle arrest in G2 and proliferation arrest.[55] KPNA2 can promote G1/S cell cycle transition[58] and is differentially expressed throughout the cell cycle, with its highest expression occurring during the G2/M phase.[59, 60] AURKA was found to cooperate with Bora to activate the kinase Plk1 and control mitotic entry.[3] Dysregulation of AURKA was found to induce abnormal G2–M transitioning in mammalian cells.[61] And finally, mutations of CDC16 and Cdc27 have been shown to lead to a defect in G2/M progression.[62] The observation that TRPS1 enriched on the promoter of CDC16 and CCNA2 not others indicates that these nine gene might be regulated by TRPS1 directly and indirectly. Further studies are required to confirmed how TRPS1 either promotes or inhibits these gene expression.

Two of these key genes regulated by TRPS1 knockdown—CDC16 and CDK5R1—showed enrichment in the G2/M stage, suggesting that TRPS1 plays an important role in the G2/M transition during cell cycle progression. We further confirmed this finding using a double thymidine assay, which showed an accumulation of cells at the G2/M stage when TRPS1 was knocked down. Since the proportions of cells in the G1 and S stages were also affected upon TRPS1 silencing, TRPS1 might also be involved in the regulation of G1 and S stages of the cell cycle.

A recent study indicates that 53BP1 functions as an inhibitor of BRCA1 accumulation at double strand breakage sites in the G1 phase of the cell cycle.[49] This indicates that 53BP1 and BRCA1 could also be involved in the TRPS1 gene regulatory network for cell cycle control. These results, together with a recent study[21] that reports that Trps1 deficiency impairs progression into metaphase during mitosis in chondrocytes, implicates TRPS1 as an important player in cell cycle control, at least in the G2 stage and the G2/M transition of the cell cycle, possibly through the control of G2/M-related gene expression.

**Figure 5:**
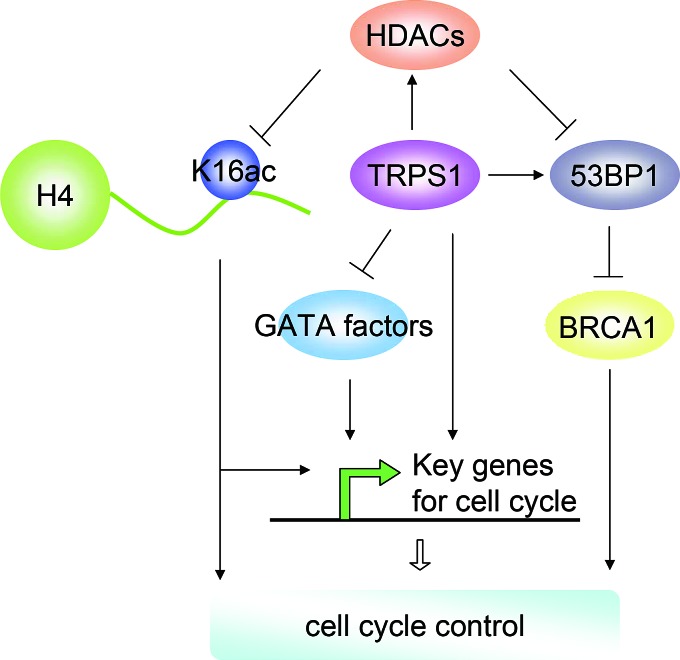
A schematic diagram depicting the potential regulatory network in which TRPS1 is implicated as a central hub in the control of the cell cycle

GATA factors play critical roles in cell-fate specification, differentiation, proliferation and migration,[63] and previous work has demonstrated that TRPS1 represses the transcriptional activation of many GATA factors.[13, 14] Although GATA factors involved in cell cycle control remain largely uncharacterized, several studies have implicated their involvement in cell cycle progression. For example, GATA-1 activity is low in G1, peaks during the mid-S phase, and then decreases in the G2/M-phase;[64] GATA-2 induction increases G(0) residency of murine and human hematopoietic cells;[65] *Gata3* knockout in mice decreases the proportion of cycling long-term hematopoietic stem cells (LTHSC);[66] and GATA4 regulates numerous cell cycle-related genes to increase cardiomyocyte proliferation.[67] In addition, GATA factors control the expression of various cell cycle genes: GATA1 inhibits *Cdk6* and cyclin D2 (*Ccdn2*) but induces the expression of the cyclin-dependent kinase inhibitors *Cdkn2c* and *Cdkn1b*;[39, 68, 69] GATA2 regulates *Cdkn2a* and *Cdkn1b*;[65, 70] GATA6 regulates *Cdkn2a*;[71] and GATA4 directly regulates *Ccdn2* and *Cdk4*.[67] Although we show the effects of TRPS1 on several cell cycle genes, it remains to be determined how or if TRPS1 represses GATA factors to control the expression of these genes. Overall, TRPS1 is likely to be involved in modulating the expression of cell cycle genes via inhibiting the transcriptional activity of the GATA factors. Figure 5 shows a schematic to suggest a mechanism by which TRPS1 could be involved in regulating GATA factors as well as these cell cycle-related genes.

DNA replication and chromatin segregation are important events during cell cycle progression and are dependent on DNA-histone interactions, which are regulated by histone-modifying enzymes, such as histone acetyltransferases (HATs) and histone deacetylases (HDACs).[4] It has been reported that impaired HDAC function affects the mitotic checkpoint and induces cell cycle arrest at the prometaphase.[72, 73] We found a decrease in HDAC activity and HDAC2 and HDAC4 expression but an increase in histone H4K16 acetylation after TRPS1 silencing, suggesting that TRPS1 regulates HDAC expression and activity to modulate histone modification, another mechanism by which TRPS1 controls cell cycle progression (Figure 5). HDACs are known to play crucial roles in numerous biological processes largely through their repressive influence on transcription.[4] Thus, TRPS1 might also control the expression of key cell cycle genes by modulating histone acetylation through the control of HDAC expression and activity (Figure 5).

TRPS1 was found to be amplified in SK-Br-3 breast cancer cell lines[74] as well as in ZR-75-1, T-47D and MCF7 cell lines,[35] and in >90% of breast cancers.[38] In contrast, MDA-MB-231 breast cancer cells exhibit low TRPS1 mRNA and protein levels. In tissue samples, higher TRPS1 expression is significantly associated with ERα+, progesterone receptor (PR)+, HER2+ expression in breast cancers, as well as improved survival.[37] TRPS1 was considered a candidate oncogene associated with ER+ HER2-amplified breast cancers.[40] However, as with the basal-like cell lines, one study shows that TRPS1 has a lower expression in basal-like breast cancers, and is associated with poorer overall survival in these subtypes.[34] Here, we found elevated *Trps1* expression in Luminal breast cancer cell lines as compared with Basal breast cancer cell lines, as well as higher expression in luminal breast cancers as compared with the other subtypes. Interestingly, Her2+ breast cancers and Claudin^low^ subtypes showed reduced expression as compared with basal breast cancers.

Several reports have provided evidence to show that TRPS1 inhibits EMT during breast cancer development.[36, 41-43] The six hallmarks of cancer—sustained proliferative signaling, evasion of growth suppressors, resistance against cell death, replicative immortality, induction of angiogenesis, and activation of invasion and metastasis[51]—endow cancer cells with a capacity for uncontrolled growth and metastasis: the two defining features of cancer. EMT is an important process in the control of metastasis, and previous studies have shown that TRPS1 modulates EMT in prostate[13, 29, 30] and breast[36, 41-43] cancers. Here we provide evidence that TRPS1 promotes the proliferation of BT474 breast cancer cells by affecting cell cycle progression. TRPS1 expression was decreased between Grade 1 and Grade 3 breast cancers and in luminal-B breast cancers. Although higher TRPS1 was claimed to be associated with improved survival,[37, 75] we found that a high expression of Trps1 leads to better survial before 18 years for overall survival(p=0.0040) and before 16 years for disease-free survival (*p*=0.0112) (Figure S3). These phenomena might, at least in part, be due to the dual role of TRPS1 as both an oncogene and a tumor suppressor during breast cancer development: The tumor suppressor role of TRPS1 might be due, to some extent, to its function as a negative regulator of EMT and the higher expression of TRPS1 could reduce the tumor's potential for metastasis. The oncogenic role of TRPS1, however, might be because of TRPS1's function as a positive regulator of cancer cell proliferation, which will lead to tumor growth. Tumor growth and metastasis are two distinct processes in carcinoma progression. TPRS1 might exert its two distinct functions at different times during tumor progression by reducing metastasis and thus delaying an earlier relapse but, at later stages, acting to promote proliferation and thereby worsening the prognosis. The mechanism(s) controlling these two antagonistic functions requires a much deeper investigation and it is intriguing to consider that the functions of TRPS1 may differ at certain developmental stages to exert its critical roles in breast tumorigenesis and progression.

In summary, our study provides evidence to show that TRPS1 is a central hub in the complex regulatory network controlling cell cycle progression. Our findings will also help to unravel the oncogenic activity of TRPS1 in cancer development aside from its known role as a tumor suppressor.

## MATERIALS AND METHODS

### Cell culture

MCF-7, BT474, and MDA-MB-231 cell lines were cultured in Dulbecco's modified essential medium (DMEM) (Life Technologies, Carlsbad, CA) supplemented with 10% FCS and 1% penicillin-streptomycin solution (Life Technologies). MCF10A cells were cultured in DMEM/F12 media with 10% horse serum, 20 ng/ml epidermal growth factor (EGF), 100 ng/ml cholera toxin, 0.01 mg/ml insulin, 500 ng/ml hydrocortisone and 1% penicillin-streptomycin solution.

### RNA interference

siRNA mixtures consisted of three siRNAs against Trps1 (Santa Cruz Biotechnology, Santa Cruz, CA) and non-silencing siRNA control (GenePharma, Shanghai, China). Cells were transfected with siRNA using Lipofectamine RNAiMAX reagent (Invitrogen, Carlsbad, CA).

### Real-time RT-PCR

Total RNA was extracted using the RNeasy kit (Qiagen, Hilden, Germany) and reverse transcription of RNA was performed using PrimeScript RT reagent kit (TaKaRa, Otsu, Shiga, Japan) according to the manufacturer's instructions. Real-time PCR reactions were performed with SYBRPremix Ex Taq (TaKaRa) in a Bio-Rad CFX96 Real-Time PCR System (Bio-rad, Hercules, CA). Endogenous β-actin was used for normalization. The primer sequences for real time RT-PCR are listed in Supplementary Table 2.

### Cell proliferation and apoptosis assay

Cell proliferation was measured with the CCK-8 kit (Dojindo Laboratories, Kumamoto, Japan) according to the protocol recommended by the manufacturer. For the cellular apoptosis assay, cells were stained using Annexin-V/Dead Cell Apoptosis Kit (Invitrogen) as per the manufacturer's recommendations and analyzed on a BD FACSCalibur flow cytometry (BD Biosciences, Franklin Lakes, NJ).

### Cell cycle analysis

For cell cycle analysis, unsynchronized cells were harvested by trypsinization and fixed with 70% ethanol. Cells were then stained with propidium iodide for total DNA content and the cell cycle distribution was then analyzed using a BD FACSCalibur flow cytometry (Becton Dickinson).

A double thymidine block was used to synchronize the cells at the G1/S stage. Briefly, cells were grown in complete medium to about 50% confluence and then washed twice in phosphate-buffered saline (PBS). Cells were then grown in complete medium supplemented with 2 mM thymidine (block medium) for 16 h to introduce the first block. The medium was then replaced with fresh complete medium to release the cells. Ten hours later, a second block was introduced by incubating the cells with block medium as before. The block medium was removed after 17 h by washing with PBS three times and the cells were then grown in fresh complete medium to allow cells to progress synchronously through the G2 and M phases. The cell cycle analysis was carried out at different time points as per the protocol above for unsynchronized cells.

### Human cell cycle PCR array

Total RNA was isolated from BT474 cells transfected with control or TRPS1 siRNA using the RNeasy Mini Kit (Qiagen) according to the manufacturer's instructions and immediately reversely transcribed using Maxima First Strand cDNA Synthesis Kit for RT-qPCR (Qiagen). A Human Cell Cycle RT^2^ Profiler PCR Array (Qiagen), containing primers for 84 tested cell cycle-related genes and 5 housekeeping genes as controls, was then used to perform real-time PCR in the Bio-Rad CFX96 Real-Time PCR System, according to the manufacturer's instructions. Data was analyzed using the RT² Profiler^TM^ PCR Array Data Analysis software (Qiagen).

### HDAC activity assay

HDAC activity assays were performed using the colorimetric HDAC activity assay from BioVision Research Products (Mountain View, CA) according to manufacturer's instructions. The relative OD values were analyzed using an iMark Microplate Absorbance Reader (Bio-Rad) at 405 nm.

### Western blot analysis

Antibodies for HDAC1–6 and 53BP1 were obtained from Cell Signaling Technology (Beverly, MA). TRPS1 antibody was purchased from R&D Systems (Minneapolis, MN). HDAC9 antibody was purchased from Abcam (Cambridge, MA). H3 and H4 antibodies were obtained from EMD Millipore (Billerica, MA). Ac-H3K9, ac-H3K18 and ac-H4K16 antibodies were purchased from Active Motif (Carlsbad, CA). P53 antibody and anti-rabbit and anti-goat secondary antibodies were purchased from Santa Cruz Biotechnology. β-actin antibody and anti-mouse secondary antibody were purchased from Proteintech (Chicago, IL). For western blotting, protein lysates were separated by SDS-PAGE, transferred to PVDF membranes, and immunoblotted with the respective antibodies as indicated above and in the figures. Blots were developed with SuperSignal West Femto Maximum Sensitivity Substrate (Pierce/Thermo Scientific, Rockford, IL).

### Co-immunoprecipitation

Co-immunoprecipitation(Co-IP) was performed using anti-TRPS1 antibody (R&D systems, Minneapolis, MN) and control polyclonal IgG (Santa Cruz Biotechnology, Santa Cruz, CA) and Dynabeads Protein G (Invitrogen, Carlsbad, CA) according to manufacturer's instructions. MS analysis and western blot were used to study the immnuoprecipitated proteins.

### Immunofluorescence

For immunofluorescence studies, BT474 were cultured on glass slides for 24 hours. Prior to staining, cells were fixed in 4% paraformaldehyde for 25 min, permeabilized in 0.1% Triton X-100 for 20 min, and blocked 30 min in 1% BSA at room temperature. Mouse monoclonal antibodies to HDAC1 (Cell Signaling Technology, Beverly, MA), HDAC2 (Cell Signaling Technology, Beverly, MA) and goat polyclonal antibody to TRPS1 (R&D Systems, Minneapolis, MN) were used as primary antibodies. Fluorescence was detected by Alexa Fluor 488 or Alexa Fluor 594-conjugated secondary antibodies (Invitrogen, Carlsbad, CA). The nuclei were stained with DAPI (Vector Laboratories, Cambridgeshire, UK). Representative images were captured using the Leica DM5000 B microscope (Leica Microsystems, Buffalo Grove, IL).

### Chromatin immunoprecipitation (ChIP) analysis

BT474 cells were washed and cross-linked with 1% formaldehyde at room temperature for 11 min. Reaction was stopped using 125 mM glycine solution. Cells were washed with PBS and lysed in lysis buffer (1% SDS, 10 mM EDTA, 50 Mm Tris-HCl pH 8.1, 1 mM PMSF, 5 mM NaF, 5 mM Na3VO4, 2 μg/ml leupeptin, 5 μg/ml aprotinin, 1 μg/ml pepstatin), followed by sonication. The chromatin was fragmented by sonification to an average size of 250bp. Supernatants were then recovered by centrifugation at 13000g for 10min at 4 °C. Then the supernatants were diluted using dilution buffer (1% Triton X-100, 2 mM EDTA, 150 mM NaCl, 20 mM Tris-HCl pH 8.0) and subjected to one round of immunoclearing for 2 h at 4 °C with 40 μl of protein G. Immunoprecipitation was performed overnight with anti-TRPS1 primary antibody (R&D Systems, Minneapolis, MN), IgG control (Santa Cruz Biotechnology, Santa Cruz, CA) and RNA polymerase II primary antibody (EMD Millipore, Billerica, MA), 40μl of protein G (Invitrogen, Carlsbad, CA) were further added and incubated for 3h at 4 °C. Immunoprecipitates were washed sequentially in RIPA buffer, RIPA-NaCl buffer, LiCl buffer. Beads precipitates were then washed once with TE buffer and eluted once with extraction buffer. Eluates were heated at 65 °C overnight reverse the formaldehyde cross-linking. DNA was precipitated using phenol/ chloroform/isoamylalcohol method. Quantitative Real-Time PCR was performed with SYBRPremix Ex Taq (TaKaRa) in a Bio-Rad CFX96 Real-Time PCR System (Bio-rad, Hercules, CA) for ChIP analysis and quantification. The well characterized untranscribed genomic regions Untr12 and Untr4 were used as negative genomic controls. Sequences of the primers used for ChIP-qPCR were listed in Supplementary table 3.

### Data preprocessing of Affymetrix microarray gene expression

The gene expression data of 51 breast cancer cell lines were retrieved from previously published work.[50] Data preprocessing of the microarray gene expression of breast cancer samples was performed as previously described.[76] Briefly, 26 breast cancer cohorts on Affymetrix U133A or U133Plus2 were downloaded from Gene Expression Omnibus (GEO) and ArrayExpress. Robust Multichip Average (RMA) normalization was performed on each cohort and the normalized data was subsequently standardized using ComBat to remove the batch effect.[77] The standardized data yielded a dataset of 3,992 breast cancer tumors and 22 normal breast tissue samples.

### Identification of breast cancer subtypes

Breast cancer subtype signature was obtained from previous work.[78] Single sample Gene Set Enrichment Analysis (ssGSEA) was computed based on the breast cancer subtype signature for each sample.[79] This analysis was then used to assign a subtype to each sample.

### Statistical analysis

Statistical analysis was performed using Matlab® R2012a (Mann-Whitney test) and Graphpad Prism® version 5.0 (Kaplan-Meier analysis and log-rank test).

## SUPPLEMENTARY MATERIAL FIGURES AND TABLES


