# Supplementary material for: A central role for TRPS1 in the control of cell cycle and cancer development
Source: Oncotarget. 2014 Jul 31;5(17):7677–90. doi: 10.18632/oncotarget.2291 (PMC4202153; doi:10.18632/oncotarget.2291)
Supplement: Supplementary file 1 [file oncotarget-05-7677-s001.pdf]

A central role for TRPS1 in the control of cell cycle and cancer development

Supplementary Material

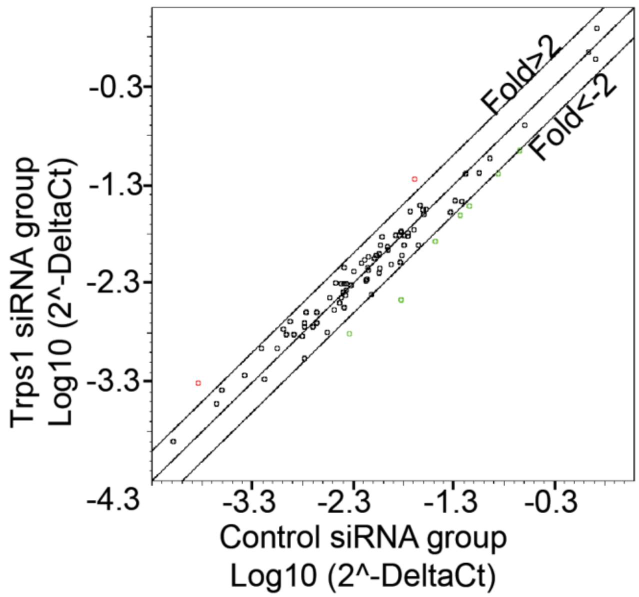

Figure S1. Scatterplot decipher the human cell cycle RT2 Profiler PCR Array data containing 84 tested cell cycle-related genes. Green and red circles indicate significantly down- and up-regulated genes, respectively.

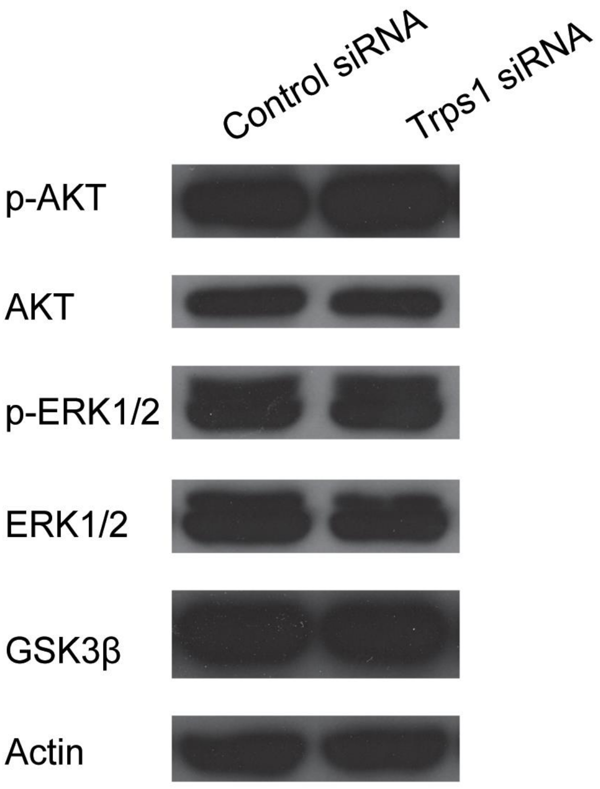

Figure S2. The effects of TRPS1 silencing on EGF-R/HER2 signaling pathway downstream targets: AKT, ERK1/2 and GSK3β.

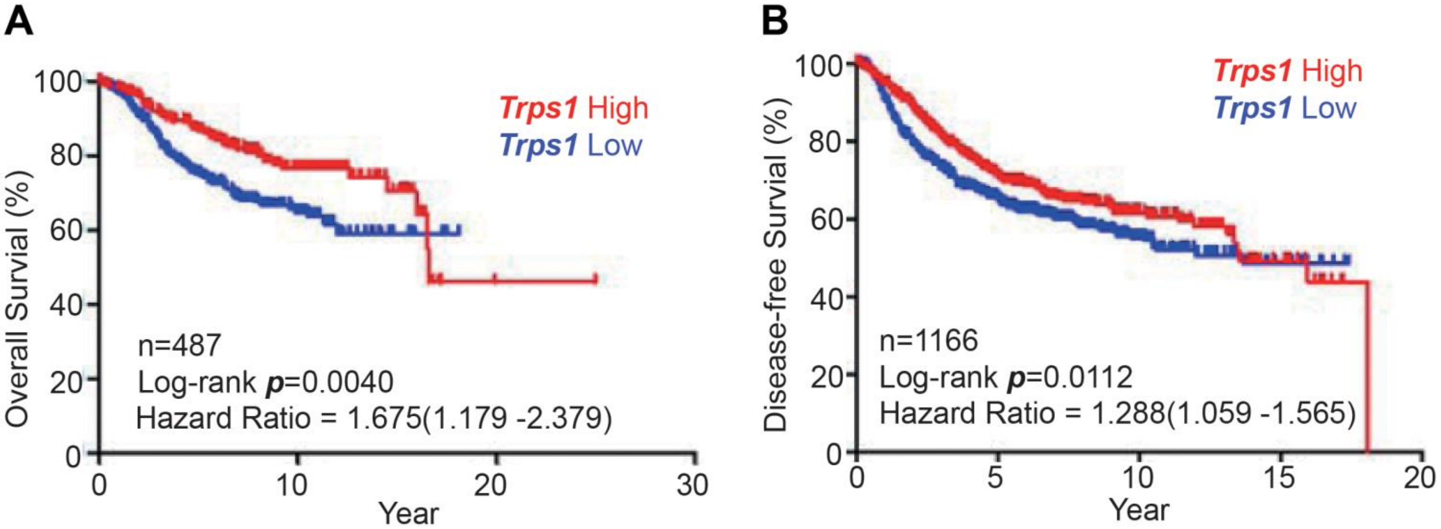

Figure S3. Prognostic value of Trps1 expression in breast cancers. (A) Comparison of overall survival of breast cancer patients with high and low Trps1 expression. (B) Comparison of disease-free survival of breast cancer patients with high and low Trps1 expression.

Supplementary Table 1. TRPS1 interactomes

Protein ID	Protein names	Gene name	PepCount	UniquePep	CoverPerce
Q9UHF7	Zinc finger transcription factor Trps1 (Tricho-r	TRPS1	74	29	28.10%
P35580	Myosin-10 (Cellular myosin heavy chain, type	MYH10	25	22	13.87%
F8W6L6	Myosin-10	MYH10	25	22	13.76%
P42166	Lamina-associated polypeptide 2, isoform alp	TMPO LAP2	29	18	32.56%
F2Z2U8	Myosin-14	MYH14	16	15	8.04%
G8JLL9	Myosin-14	MYH14	16	15	8.01%
Q7Z406	Myosin-14 (Myosin heavy chain 14) (Myosin h	MYH14 KIA	16	15	8.17%
B4DE59	cDNA FLJ60424, highly similar to Junction plakoglobin		14	12	19.72%
P42167	Lamina-associated polypeptide 2, isoforms be	TMPO LAP2	17	11	27.97%
B4DPP6	cDNA FLJ54371, highly similar to Serum albumin		30	9	13.11%
P02533	Transferrin receptor protein 1 (TR) (TfR) (TfR1	TFRC	11	9	15.68%
F5GWP8	Junction plakoglobin	JUP	10	9	12.69%
P38646	Stress-70 protein, mitochondrial (75 kDa gluc	HSPA9 GRP	9	9	14.87%
Q8N1C8	HSPA9 protein (Fragment)	HSPA9	9	9	14.83%
B7Z4V2	cDNA FLJ51907, highly similar to Stress-70 protein, mitoc		9	9	15.19%
B7Z4T3	cDNA FLJ51903, highly similar to Stress-70 protein, mitoc		9	9	15.98%
P51114	Fragile X mental retardation syndrome-relate	FXR1	10	8	12.56%
B4DXZ6	Fragile X mental retardation syndrome-relate	FXR1	10	8	12.83%
B1PS43	Myosin heavy chain 11 smooth muscle isoform	MYH11	9	8	4.34%
P35749	Myosin-11 (Myosin heavy chain 11) (Myosin h	MYH11 KIA	9	8	4.46%
B4DZW4	cDNA FLJ57246, highly similar to Poly(A)-binding protein 1		10	7	11.51%
E7EQV3	Polyadenylate-binding protein 1	PABPC1	10	7	11.51%
P11940	Polyadenylate-binding protein 1 (PABP-1) (Pol	PABPC1 PA	10	7	10.69%
B4DM78	cDNA FLJ58199, highly similar to Fragile X mental retarda		9	7	12.92%
Q7Z417	Nuclear fragile X mental retardation-interacti	NUFIP2 KIA	8	7	12.95%
A1L3A7	Nuclear fragile X mental retardation protein ir	NUFIP2 hC	8	7	12.95%
P09874	Poly [ADP-ribose] polymerase 1 (PARP-1) (EC 2	PARP1 ADP	7	7	7.50%
B2R5W3	cDNA, FLJ92658, highly similar to Homo sapiens poly (ADI		7	7	7.50%
P11021	78 kDa glucose-regulated protein (GRP-78) (E	HSPA5 GRP	7	7	12.08%
Q8NC56	LEM domain-containing protein 2 (hLEM2)	LEMD2	6	6	11.33%
Q8WWM7	Ataxin-2-like protein (Ataxin-2 domain protein	ATXN2L A2	6	6	5.02%
H3BUF6	Ataxin-2-like protein	ATXN2L	6	6	5.06%
Q96HS1	Serine/threonine-protein phosphatase PGAM	PGAM5	6	6	17.65%
B2RDN9	cDNA, FLJ96699, highly similar to Homo sapiens thyroid a		6	6	10.18%
B4DE32	cDNA FLJ53970, highly similar to ATP-dependent DNA hel		6	6	11.09%
B1AHC9	X-ray repair cross-complementing protein 6	XRCC6	6	6	11.09%
P12956	X-ray repair cross-complementing protein 6 (E	XRCC6 G22	6	6	10.18%
Q04837	Single-stranded DNA-binding protein, mitoch	SSBP1 SSBF	7	5	37.84%
A4D1U3	Single-stranded DNA-binding protein	SSBP1 hCG	7	5	37.84%
Q567R6	Single-stranded DNA-binding protein	SSBP1	7	5	37.84%
P07437	Tubulin beta chain (Tubulin beta-5 chain)	TUBB TUBE	6	5	14.41%
B4DY90	cDNA FLJ56903, highly similar to Tubulin beta-7 chain		6	5	13.79%
Q5JP53	Tubulin beta chain	TUBB	6	5	15.02%
B7ZAF0	cDNA, FLJ79164, highly similar to Tubulin beta-7 chain		6	5	15.42%
Q5SU16	Beta 5-tubulin (Beta-tubulin protein) (Tubulin	TUBB OK/S	6	5	14.41%
P08779	Keratin, type I cytoskeletal 16 (Cytokeratin-16	KRT16 KRT	6	5	8.88%
Q13310	Polyadenylate-binding protein 4 (PABP-4) (Pol	PABPC4 AP	6	5	7.92%
B1ANR0	Poly(A) binding protein, cytoplasmic 4 (Inducil	PABPC4 hC	6	5	8.29%

Q6IQ30	PABPC4 protein	PABPC4	6	5	7.73%
P50402	Emerin	EMD EDMD	5	5	19.29%
Q00059	Transcription factor A, mitochondrial (mtTFA)	TFAM TCF6	5	4	13.41%
E5KSU5	Mitochondrial transcription factor A (Transcri	TFAM hCG_	5	4	13.41%
H7BYN3	Transcription factor A, mitochondrial (Fragme	TFAM	5	4	15.07%
E5KSX8	Mitochondrial transcription factor A		5	4	13.41%
Q6LES8	TFAM protein (Fragment)	TFAM	5	4	13.41%
J3QRK5	Protein UBBP4	UBBP4	5	4	16.16%
P27694	Replication protein A 70 kDa DNA-binding sub	RPA1 REPA	4	4	6.82%
P46781	40S ribosomal protein S9	RPS9	4	4	16.49%
A5D904	RPS9 protein (Fragment)	RPS9	4	4	28.32%
B5MCT8	40S ribosomal protein S9 (Ribosomal protein S	RPS9 hCG_	4	4	23.02%
B7Z732	cDNA FLJ61636, highly similar to 40S ribosomal protein S9		4	4	20.38%
C9JM19	40S ribosomal protein S9 (Ribosomal protein S	RPS9 hCG_	4	4	20.51%
P51116	Fragile X mental retardation syndrome-relate	FXR2 FMR1	4	4	5.94%
Q08211	ATP-dependent RNA helicase A (RHA) (EC 3.6.	DHX9 DDX9	4	4	3.15%
Q09666	Neuroblast differentiation-associated protein	AHNAK PM	4	4	0.80%
Q92499	ATP-dependent RNA helicase DDX1 (EC 3.6.4.1	DDX1	4	4	4.59%
B4DPN6	cDNA FLJ51031, highly similar to ATP-dependent RNA hel		4	4	5.47%
A3RJH1	ATP-dependent RNA helicase DDX1 (DEAD (As	DDX1 hCG_	4	4	4.59%
E5KLB5	DNA ligase (EC 6.5.1.1)		4	4	4.16%
E5KLB6	DNA ligase (EC 6.5.1.1)	LIG3 hCG_2	4	4	4.43%
P49916	DNA ligase 3 (EC 6.5.1.1) (DNA ligase III) (Poly	LIG3	4	4	4.16%
K7EQB6	60S ribosomal protein L27	RPL27 hCG_	4	4	22.70%
K7ERZ5	Histone H3.3 (Fragment)	H3F3B	4	4	7.71%
B7Z6I3	DNA ligase (EC 6.5.1.1)		4	4	5.69%
J3QRS3	Myosin regulatory light chain 12A (Myosin reg	MYL12A M	4	4	23.16%
Q53HL1	Myosin regulatory light chain MRCL3 variant (Fragment)		4	4	23.98%
O14950	Protein transport protein Sec16A (SEC16 hom	SEC16A KIA	4	4	23.84%
P19105	Myosin regulatory light chain 12A (Epididymis	MYL12A M	4	4	23.98%
M0QZC5	Inactive serine/threonine-protein kinase VRK3	VRK3	4	4	27.97%
P62280	40S ribosomal protein S11	RPS11	4	4	20.89%
Q8IY44	CTBP2 protein	CTBP2	4	4	6.63%
P56545	C-terminal-binding protein 2 (CtBP2)	CTBP2	4	4	7.64%
Q5SQP8	C-terminal-binding protein 2	CTBP2	4	4	6.63%
Q6PIK1	IGL@ protein	IGL@	5	3	8.94%
Q71UI9	Histone H2A.V (H2A.F/Z)	H2AFV H2A	4	3	37.50%
P0C0S5	Histone H2A.Z (H2A/z)	H2AFZ H2A	4	3	37.50%
C9J0D1	Histone H2A	H2AFV	4	3	39.34%
Q8IWP6	Class IVb beta tubulin		4	3	8.31%
Q8IZ29	Tubulin, beta 2C	TUBB2C	4	3	8.31%
Q8N6N5	Tubulin, beta 2C	TUBB2C	4	3	8.31%
B3KML9	cDNA FLJ11352 fis, clone HEMBA1000020, highly similar t		4	3	9.32%
P68371	Tubulin beta-4B chain (Tubulin beta-2 chain) (	TUBB4B TU	4	3	8.31%
O94776	Heat shock 70 kDa protein 4L (Heat shock 70-I	HSPA4L AP	3	3	4.49%
P04792	Non-histone chromosomal protein HMG-14 (H	HMGN1 HN	3	3	17.07%
P06748	Nucleophosmin (NPM) (Nucleolar phosphopro	NPM1 NPM	3	3	8.50%
A4ZU86	Truncated nucleolar phosphoprotein B23	NPM1	3	3	9.12%
P13010	X-ray repair cross-complementing protein 5 (E	XRCC5 G22	3	3	4.64%
Q53T09	Putative uncharacterized protein XRCC5 (Frag	XRCC5	3	3	5.99%

P16402	Histone H1.3 (Histone H1c) (Histone H1s-2)	HIST1H1D I	3	3	13.12%
P16403	Histone H1.2 (Histone H1c) (Histone H1d) (His	HIST1H1C H	3	3	13.62%
P10412	Histone H1.4 (Histone H1b) (Histone H1s-4)	HIST1H1E H	3	3	13.24%
A3R0T7	Liver histone H1e		3	3	13.24%
A3R0T8	Histone 1, H1e (Histone H1e)	HIST1H1E H	3	3	13.24%
Q4VB24	Histone cluster 1, H1e	HIST1H1E	3	3	13.24%
B2R984	cDNA, FLJ94268, highly similar to Homo sapiens histone 1		3	3	13.24%
P16989	Y-box-binding protein 3 (Cold shock domain-c	YBX3 CSDA	3	3	13.71%
P20700	Lamin-B1	LMNB1 LM	3	3	4.95%
P22626	Heterogeneous nuclear ribonucleoproteins A2	HNRNPA2B	3	3	9.92%
P52272	Heterogeneous nuclear ribonucleoprotein M (	HNRNPM H	3	3	3.70%
P62269	40S ribosomal protein S18 (Ke-3) (Ke3)	RPS18 D6S2	3	3	15.79%
P78527	DNA-dependent protein kinase catalytic subu	PRKDC HYR	3	3	0.85%
E7EUY0	DNA-dependent protein kinase catalytic subu	PRKDC	3	3	0.85%
Q07955	Serine/arginine-rich splicing factor 1 (Alternat	SRSF1 ASF S	3	3	10.89%
Q59FA2	Splicing factor, arginine/serine-rich 1 (Splicing factor 2, alt		3	3	11.59%
J3KTL2	Serine/arginine-rich-splicing factor 1 (Splicing	SRSF1 SFRS	3	3	10.67%
Q13363	C-terminal-binding protein 1 (CtBP1) (EC 1.1.1	CTBP1 CTBI	3	3	5.91%
Q4KMQ8	C-terminal binding protein 1	CTBP1	3	3	5.91%
A8K486	Peptidyl-prolyl cis-trans isomerase (EC 5.2.1.8)		3	3	17.58%
Q71V99	Peptidyl-prolyl cis-trans isomerase (EC 5.2.1.8)		3	3	17.68%
B2RE56	Peptidyl-prolyl cis-trans isomerase (EC 5.2.1.8)		3	3	17.58%
P62937	Peptidyl-prolyl cis-trans isomerase A (PPIase A	PPIA CYPA	3	3	17.58%
B2R491	Ribosomal protein S4, X-linked, isoform CRA_c	RPS4X hCG	3	3	7.22%
Q53HV1	Ribosomal protein S4, X-linked X isoform variant (Fragme		3	3	7.22%
Q96IR1	RPS4X protein (Fragment)	RPS4X	3	3	7.82%
P62701	40S ribosomal protein S4, X isoform (SCR10) (S	RPS4X CCG	3	3	7.22%
B4DUQ1	cDNA FLJ54552, highly similar to Heterogeneous nuclear r		3	3	8.43%
Q6IBN1	HNRPK protein (Heterogeneous nuclear ribon	HNRPK HNI	3	3	7.97%
Q5EC54	Heterogeneous nuclear ribonucleoprotein K tr	HNRPK	3	3	7.97%
P61978	Heterogeneous nuclear ribonucleoprotein K (h	HNRNPK HI	3	3	7.99%
Q5T6W5	Heterogeneous nuclear ribonucleoprotein K	HNRNPK	3	3	8.64%
E7EPN9	Protein PRRC2C	PRRC2C	3	3	1.14%
Q9Y520	Protein PRRC2C (BAT2 domain-containing pro	PRRC2C BA	3	3	1.10%
H0Y449	Nuclease-sensitive element-binding protein 1	YBX1	3	3	12.30%
P67809	Nuclease-sensitive element-binding protein 1	YBX1 NSEP1	3	3	14.20%
Q6PKI6	YBX1 protein (Fragment)	YBX1	3	3	17.29%
Q05D43	YBX1 protein (Fragment)	YBX1	3	3	17.23%
Q2VIK8	RcNSEP1 (Fragment)		3	3	14.42%
H0Y4R1	Inosine-5'-monophosphate dehydrogenase 2 (	IMPDH2	3	3	8.30%
P12268	Inosine-5'-monophosphate dehydrogenase 2 (	IMPDH2 IM	3	3	7.59%
J3KPS3	Fructose-bisphosphate aldolase (EC 4.1.2.13)	ALDOA hCG	3	3	8.42%
P04075	Heat shock protein beta-1 (HspB1) (28 kDa he	HSPB1 HSP	3	3	8.52%
H3BQN4	Fructose-bisphosphate aldolase (EC 4.1.2.13)	ALDOA	3	3	8.59%
S6BGD6	IgG L chain		3	3	9.79%
Q6IN99	IGL@ protein	IGL@	3	3	9.79%
Q6GMX3	IGL@ protein	IGL@	3	3	9.75%
Q567P1	IGL@ protein	IGL@	3	3	9.79%
Q8NEJ1	Uncharacterized protein		3	3	9.75%
O75531	Metastasis-associated protein MTA2 (Metasta	MTA2 MTA	5	2	33.71%

Q59GP5	Eukaryotic translation elongation factor 1 alpha 2 variant		3	2	5.59%
P14625	Endoplasmin (94 kDa glucose-regulated prote	HSP90B1 G	2	2	3.24%
Q59FC6	Tumor rejection antigen (Gp96) 1 variant (Fragment)		2	2	4.51%
Q5CAQ5	Tumor rejection antigen (Gp96) 1	TRA1	2	2	3.24%
P16401	Histone H1.5 (Histone H1a) (Histone H1b) (His	HIST1H1B H	2	2	9.29%
P18124	60S ribosomal protein L7	RPL7	2	2	8.47%
P18887	DNA repair protein XRCC1 (X-ray repair cross-	XRCC1	2	2	2.69%
F5H8D7	DNA repair protein XRCC1	XRCC1	2	2	2.82%
Q59HH7	X-ray repair cross complementing protein 1 variant (Fragr		2	2	2.63%
B4DEB2	cDNA FLJ56491, highly similar to DNA-repair protein XRC(		2	2	4.25%
P19338	Nucleolin (Protein C23)	NCL	2	2	3.24%
B3KTP9	cDNA FLJ38578 fis, clone HCHON2007674, highly similar t		2	2	3.63%
B3KM80	Nucleolin, isoform CRA_c (cDNA FLJ10452 fis,	NCL hCG_3	2	2	4.29%
Q6ZS99	cDNA FLJ45706 fis, clone FEBRA2028457, highly similar to		2	2	3.81%
P26599	Polypyrimidine tract-binding protein 1 (PTB) (!	PTBP1 PTB	2	2	3.58%
P49792	E3 SUMO-protein ligase RanBP2 (EC 6.3.2.-) (3	RANBP2 NU	2	2	0.87%
P62263	40S ribosomal protein S14	RPS14 PRO	2	2	15.89%
P62277	40S ribosomal protein S13	RPS13	2	2	11.26%
P62854	40S ribosomal protein S26	RPS26	2	2	20.87%
Q75MH1	HCG1745083 (Putative uncharacterized prote	RPS26 LOC	2	2	20.87%
P63173	60S ribosomal protein L38	RPL38	2	2	24.29%
Q06830	Peroxiredoxin-1 (EC 1.11.1.15) (Natural killer (	PRDX1 PAG	2	2	9.05%
B2R4P2	cDNA, FLJ92164, highly similar to Homo sapiens peroxire(		2	2	9.05%
Q14694	Ubiquitin carboxyl-terminal hydrolase 10 (EC 3	USP10 KIAA	2	2	3.76%
Q92900	Regulator of nonsense transcripts 1 (EC 3.6.4.	UPF1 KIAA(	2	2	1.95%
Q9Y3I0	tRNA-splicing ligase RtcB homolog (EC 6.5.1.3)	RTCB C22or	2	2	2.97%
A8MQB8	Fragile X mental retardation protein 1	FMR1	2	2	2.75%
R9WNI0	Fragile X mental retardation 1	FMR1	2	2	2.94%
Q06787	Fragile X mental retardation protein 1 (FMRP)	FMR1	2	2	2.53%
G3V0J0	Fragile X mental retardation 1, isoform CRA_e	FMR1 hCG_	2	2	2.70%
G8JL90	Fragile X mental retardation 1 isoform B (Frag	FMR1	2	2	2.62%
R9WQY1	Fragile X mental retardation 1	FMR1	2	2	2.83%
Q59GC1	Fragile X mental retardation 1 variant (Fragment)		2	2	3.04%
B1AKN7	Nuclear factor 1	NFIA	2	2	4.94%
B1AKN5	Nuclear factor 1	NFIA	2	2	4.72%
B1AKN8	Nuclear factor 1	NFIA	2	2	4.32%
Q12857	Nuclear factor 1 A-type (NF1-A) (Nuclear facto	NFIA KIAA1	2	2	4.52%
B4DRN9	Nuclear factor 1		2	2	4.52%
B4DLW8	Probable ATP-dependent RNA helicase DDX5 (	DDX5	2	2	3.36%
B4DN41	cDNA FLJ53366, highly similar to Probable ATP-dependen		2	2	2.99%
P17844	Probable ATP-dependent RNA helicase DDX5 (	DDX5 G17P	2	2	2.93%
J3KTA4	Probable ATP-dependent RNA helicase DDX5	DDX5	2	2	2.93%
B4DMJ6	cDNA FLJ50996, highly similar to 60S ribosomal protein L4		2	2	4.11%
P36578	60S ribosomal protein L4 (60S ribosomal prote	RPL4 RPL1	2	2	3.98%
Q59GY2	Ribosomal protein L4 variant (Fragment)		2	2	3.85%
H3BM89	60S ribosomal protein L4 (Ribosomal protein I	RPL4 hCG_	2	2	5.11%
B4DQN8	cDNA FLJ50056, highly similar to Homo sapiens BAT2 dom		2	2	2.33%
B4DZQ7	cDNA FLJ58652, highly similar to Probable ATP-dependen		2	2	5.48%
Q59F66	DEAD box polypeptide 17 isoform p82 variant (Fragment)		2	2	3.12%
H3BLZ8	Probable ATP-dependent RNA helicase DDX17	DDX17	2	2	3.15%

C9JMU5	Probable ATP-dependent RNA helicase DDX17	DDX17	2	2	3.53%
Q92841	Probable ATP-dependent RNA helicase DDX17	DDX17	2	2	3.16%
B4E380	Histone H3		2	2	12.39%
K7ES00	Alternative protein ADCY1	ADCY1	2	2	9.27%
K7EMV3	Histone H3	H3F3B	2	2	15.22%
Q16695	Histone H3.1t (H3/t) (H3t) (H3/g)	HIST3H3 H3	2	2	10.29%
B4DEB1	Histone H3	H3F3A	2	2	11.38%
P68431	Histone H3.1 (Histone H3/a) (Histone H3/b) (H	HIST1H3A H	2	2	10.29%
Q71DI3	Histone H3.2 (Histone H3/m) (Histone H3/o)	HIST2H3A;	2	2	10.29%
B7Z4T6	Nuclear factor 1		2	2	4.33%
P08651	Nuclear factor 1 C-type (NF1-C) (Nuclear facto	NFIC NFI	2	2	4.33%
Q6FI30	Nuclear factor 1	NFIC	2	2	4.41%
B7Z5E7	cDNA FLJ51046, highly similar to 60 kDa heat shock prote		2	2	4.06%
B7Z597	cDNA FLJ54373, highly similar to 60 kDa heat shock prote		2	2	3.72%
P10809	60 kDa heat shock protein, mitochondrial (60	HSPD1 HSP	2	2	3.66%
B3GQS7	Mitochondrial heat shock 60kD protein 1 varia	HSPD1	2	2	3.69%
B7Z4F6	cDNA FLJ54912, highly similar to 60 kDa heat shock prote		2	2	3.82%
B7ZW41	Heterogeneous nuclear ribonucleoprotein C-li	LOC440563	2	2	6.48%
A8K9A4	cDNA FLJ75154, highly similar to Homo sapiens heteroger		2	2	6.21%
Q6PKD2	HNRPCL1 protein (Fragment)	HNRPCL1	2	2	9.18%
G3V4W0	Heterogeneous nuclear ribonucleoproteins C1	HNRNPC	2	2	7.25%
B2R5W2	cDNA, FLJ92657, highly similar to Homo sapiens heteroge		2	2	6.55%
P07910	Heterogeneous nuclear ribonucleoproteins C1	HNRNPC HI	2	2	6.21%
O60812	Transcriptional adapter 3 (ADA3 homolog) (hA	TADA3 ADA	2	2	6.48%
G3V576	Heterogeneous nuclear ribonucleoproteins C1	HNRNPC	2	2	8.23%
B2RXH8	Heterogeneous nuclear ribonucleoprotein C-li	LOC440563	2	2	6.48%
B7ZW38	LOC649330 protein	LOC649330	2	2	6.48%
G3V4C1	Heterogeneous nuclear ribonucleoproteins C1	HNRNPC	2	2	6.51%
G3V2Q1	Heterogeneous nuclear ribonucleoproteins C1	HNRNPC	2	2	6.23%
B4DY08	Heterogeneous nuclear ribonucleoproteins C1	HNRNPC	2	2	6.60%
B2R603	cDNA, FLJ92712, highly similar to Homo sapiens heteroge		2	2	6.27%
D6REM6	Matrin-3	MATR3	2	2	3.53%
Q68D11	Putative uncharacterized protein DKFZp686K2	DKFZp686K	2	2	3.31%
A8MXP9	Matrin-3	MATR3	2	2	3.13%
P43243	Matrin-3	MATR3 KIA	2	2	3.31%
Q5ZEY3	Glyceraldehyde-3-phosphate dehydrogenase	GAPD	2	2	25.58%
F8VPI7	Double-stranded RNA-binding protein Staufer	STAU2	2	2	5.43%
Q9NUL3	Double-stranded RNA-binding protein Staufer	STAU2	2	2	4.56%
E7EPX0	Double-stranded RNA-binding protein Staufer	STAU2	2	2	5.30%
E7EVI1	Double-stranded RNA-binding protein Staufer	STAU2	2	2	6.67%
B7Z1P6	cDNA FLJ57953, highly similar to Double-stranded RNA-bi		2	2	6.67%
E7EVJ4	Double-stranded RNA-binding protein Staufer	STAU2	2	2	5.09%
A8K276	cDNA FLJ78317, highly similar to Homo sapiens staufen, F		2	2	5.43%
E9PH62	Double-stranded RNA-binding protein Staufer	STAU2	2	2	4.83%
E5RJN7	Double-stranded RNA-binding protein Staufer	STAU2	2	2	8.13%
Q4LE57	STAU2 variant protein (Fragment)	STAU2 varia	2	2	5.31%
Q2TBD5	Staufen, RNA binding protein, homolog 2 (Dro	STAU2 hCG	2	2	5.43%
H0YH81	ATP synthase subunit beta (EC 3.6.3.14) (Fragı	ATP5B	2	2	8.56%
F8VPV9	ATP synthase subunit beta (EC 3.6.3.14)	ATP5B	2	2	5.98%
Q0QEN7	ATP synthase subunit beta (EC 3.6.3.14) (Fragı	ATP5B	2	2	6.97%

P06576	Alpha-enolase (EC 4.2.1.11) (2-phospho-D-gly	ENO1 ENO:	2	2	5.86%
H0YHA7	60S ribosomal protein L18 (Fragment)	RPL18	2	2	14.37%
Q0QEW2	60S ribosomal protein L18 (Fragment)	RPL18	2	2	14.63%
G3V203	60S ribosomal protein L18	RPL18 hCG_	2	2	14.63%
J3QQ67	60S ribosomal protein L18 (Fragment)	RPL18	2	2	12.63%
Q07020	60S ribosomal protein L18	RPL18	2	2	12.77%
H7C4S4	Fragile X mental retardation syndrome-relate	FXR1	2	2	7.69%
Q5RLJ0	CLE		2	2	7.79%
Q549M8	CLE7 (Chromosome 14 open reading frame 16	C14orf166	2	2	7.79%
Q9Y224	UPF0568 protein C14orf166 (CLE7 homolog) (	C14orf166	2	2	7.79%
Q5U0P9	Purine-rich element binding protein A		2	2	4.35%
Q00577	Transcriptional activator protein Pur-alpha (P	PURA PUR1	2	2	4.35%
Q2NLD4	PURA protein (Fragment)	PURA	2	2	4.91%
Q56A79	Purine-rich element binding protein A	PURA	2	2	4.35%
Q2NLC9	PURA protein (Fragment)	PURA	2	2	4.67%
Q5VVC8	60S ribosomal protein L11 (Fragment)	RPL11	2	2	10.92%
Q5VVD0	Ribosomal protein L11, isoform CRA_b (cDNA,	RPL11 hCG_	2	2	10.67%
P62913	60S ribosomal protein L11 (CLL-associated an	RPL11	2	2	10.67%
Q08ES8	Cell growth-inhibiting protein 34		2	2	10.73%
Q5VVC9	60S ribosomal protein L11 (Fragment)	RPL11	2	2	14.50%
Q6FI03	G3BP protein	G3BP	2	2	6.22%
F5H4D6	Ras GTPase-activating protein-binding protein	G3BP1	2	2	10.21%
Q32P45	GTPase activating protein (SH3 domain) bindi	G3BP1	2	2	6.22%
Q13283	Ras GTPase-activating protein-binding protein	G3BP1 G3B	2	2	6.22%
B7Z8K4	cDNA FLJ51772, highly similar to Ras-GTPase-activating p		2	2	10.21%
Q5U0Q1	Putative uncharacterized protein DKFZp686L1	DKFZp686L	2	2	6.22%
Q6N092	Putative uncharacterized protein DKFZp686K1	DKFZp686K	2	2	2.70%
Q6PJX3	STAU1 protein (Fragment)	STAU1	2	2	10.71%
O95793	Ig kappa chain V-III region NG9 (Fragment)		2	2	3.64%
Q5JW28	Double-stranded RNA-binding protein Staufer	STAU1	2	2	10.55%
Q59F99	Staufen isoform b variant (Fragment)		2	2	3.55%
Q5JW30	Double-stranded RNA-binding protein Staufer	STAU1	2	2	4.25%
A8K622	cDNA FLJ75871, highly similar to Homo sapiens staufen, F		2	2	3.64%
Q8NF17	FLJ00385 protein (Fragment)	FLJ00385	2	2	3.34%
Q8WUW7	Pyruvate kinase (EC 2.7.1.40) (Fragment)	PKM2	2	2	5.83%
P14618	Pyruvate kinase PKM (EC 2.7.1.40) (Cytosolic t	PKM OIP3 F	2	2	3.77%
B4DRT3	Pyruvate kinase (EC 2.7.1.40)		2	2	3.91%
B4DNK4	Pyruvate kinase (EC 2.7.1.40)		2	2	4.38%
Q504U3	Pyruvate kinase (EC 2.7.1.40)	PKM2 PKM	2	2	5.46%
H3BTN5	Pyruvate kinase (EC 2.7.1.40) (Fragment)	PKM	2	2	4.12%
P05976	Ig kappa chain V-IV region (Fragment)	IGKV4-1	2	1	6.19%
P08590	Myosin light chain 3 (Cardiac myosin light cha	MYL3	2	1	6.15%
Q53R15	Putative uncharacterized protein MYL1 (Fragn	MYL1	2	1	8.57%
B5BU24	14-3-3 protein beta/alpha	YWHAB	2	1	4.47%
P31946	14-3-3 protein beta/alpha (Protein 1054) (Pro	YWHAB	2	1	4.47%
E5RI35	Acyl-protein thioesterase 1 (Fragment)	LYPLA1	2	1	10.00%
C9J069	Uncharacterized protein C9orf172	C9orf172	1	1	0.92%
O75528	Barrier-to-autointegration factor (Breakpoint	BANF1 BAF	1	1	1.62%
A8K899	cDNA FLJ77724, highly similar to Homo sapiens transcript		1	1	1.62%
P01621	Ig kappa chain V-IV region Len		1	1	9.00%

P02786	Fructose-bisphosphate aldolase A (EC 4.1.2.1	ALDOA ALD	1	1	1.32%
G3V0E5	Transferrin receptor (P90, CD71), isoform CRA	TFRC hCG_	1	1	1.47%
A8K6Q8	cDNA FLJ75881, highly similar to Homo sapiens transferri		1	1	1.32%
P07237	Protein disulfide-isomerase (PDI) (EC 5.3.4.1)	P4HB ERBA	1	1	1.97%
B4DLN6	cDNA FLJ59033, highly similar to Protein disulfide-isomer		1	1	2.22%
B4DNL5	Protein disulfide-isomerase (EC 5.3.4.1)		1	1	2.03%
B3KTQ9	Procollagen-proline, 2-oxoglutarate 4-dioxyge	P4HB hCG_	1	1	5.41%
B4DUA5	cDNA FLJ59430, highly similar to Protein disulfide-isomer		1	1	2.21%
H7BZ94	Protein disulfide-isomerase	P4HB	1	1	2.16%
F5H8J2	Uncharacterized protein	P4HB	1	1	2.22%
H0Y3Z3	Protein disulfide-isomerase (Fragment)	P4HB	1	1	3.65%
Q96C96	P4HB protein (Fragment)	P4HB	1	1	3.66%
P07339	Cathepsin D (EC 3.4.23.5) [Cleaved into: Cathe	CTSD CPSD	1	1	1.94%
H7C1V0	Cathepsin D light chain (Fragment)	CTSD	1	1	4.23%
P09488	Glutathione S-transferase Mu 1 (EC 2.5.1.18) (	GSTM1 GST	1	1	2.75%
P28161	Glutathione S-transferase Mu 2 (EC 2.5.1.18) (	GSTM2 GST	1	1	2.75%
Q5T8R2	Glutathione S-transferase M5, isoform CRA_b	GSTM5 hC(	1	1	2.75%
Q0D2I8	Glutathione S-transferase mu 2 (Muscle)	GSTM2	1	1	2.75%
Q03013	Glutathione S-transferase Mu 4 (EC 2.5.1.18) (	GSTM4	1	1	2.75%
P46439	Glutathione S-transferase Mu 5 (EC 2.5.1.18) (	GSTM5	1	1	2.75%
B9ZVX7	Glutathione S-transferase Mu 1	GSTM1	1	1	4.03%
E7EWW9	Glutathione S-transferase Mu 1	GSTM1	1	1	2.53%
E9PHN7	Glutathione S-transferase Mu 2	GSTM2	1	1	3.35%
Q5T8R1	Glutathione S-transferase Mu 5	GSTM5	1	1	2.53%
P11388	DNA topoisomerase 2-alpha (EC 5.99.1.3) (DN	TOP2A TOF	1	1	0.52%
P12235	ADP/ATP translocase 1 (ADP,ATP carrier prote	SLC25A4 AI	1	1	2.68%
P05141	60S acidic ribosomal protein P0 (60S ribosom	RPLP0	1	1	2.68%
P12236	ADP/ATP translocase 3 (ADP,ATP carrier prote	SLC25A6 AI	1	1	2.68%
Q6NVC0	SLC25A5 protein (Fragment)	SLC25A5	1	1	2.48%
I7HJJ0	ADP/ATP translocase 3 (Fragment)	SLC25A6	1	1	5.06%
Q9H0C2	ADP/ATP translocase 4 (ADP,ATP carrier prote	SLC25A31 /	1	1	2.54%
Q6I9V5	SLC25A6 protein (Solute carrier family 25 (Mit	SLC25A6 h(	1	1	2.68%
A8K787	cDNA FLJ75273, highly similar to Homo sapiens solute car		1	1	2.68%
Q59EI9	ADP,ATP carrier protein, liver isoform T2 variant (Fragme		1	1	2.48%
P18077	60S ribosomal protein L35a (Cell growth-inhib	RPL35A GI(	1	1	6.36%
F8WB72	60S ribosomal protein L35a	RPL35A	1	1	12.28%
F8WBS5	60S ribosomal protein L35a	RPL35A	1	1	12.73%
C9K025	60S ribosomal protein L35a (Fragment)	RPL35A	1	1	7.45%
P18621	60S ribosomal protein L17 (60S ribosomal pro	RPL17	1	1	3.80%
J3QQT2	60S ribosomal protein L17 (Fragment)	RPL17	1	1	4.09%
J3QS96	60S ribosomal protein L17 (Fragment)	RPL17	1	1	5.07%
J3QLC8	60S ribosomal protein L17	RPL17	1	1	4.02%
J3KSJ0	60S ribosomal protein L17 (Fragment)	RPL17	1	1	12.50%
J3KRB3	60S ribosomal protein L17 (Fragment)	RPL17	1	1	6.60%
H0YMT1	Talin-2 (Fragment)	TLN2	1	1	0.48%
G1UI21	Talin-2	TLN2	1	1	0.44%
J3KRX5	60S ribosomal protein L17 (Fragment)	RPL17	1	1	4.02%
Q9Y4G6	Talin-2	TLN2 KIAA(	1	1	0.28%
P22061	Protein-L-isoaspartate(D-aspartate) O-methyl	PCMT1	1	1	3.96%
B7Z972	Protein-L-isoaspartate(D-aspartate) O-methyl	PCMT1	1	1	4.69%

H7BY58	Protein-L-isoaspartate(D-aspartate) O-methyl	PCMT1	1	1	3.15%
P25705	ATP synthase subunit alpha, mitochondrial	ATP5A1 AT	1	1	1.45%
K7ENP3	60S ribosomal protein L22 (Fragment)	RPL22	1	1	1.51%
P28070	Proteasome subunit beta type-4 (EC 3.4.25.1)	PSMB4 PRC	1	1	3.03%
P30050	60S ribosomal protein L12	RPL12	1	1	4.24%
Q59FI9	Ribosomal protein L12 variant (Fragment)		1	1	3.55%
P35251	Replication factor C subunit 1 (Activator 1 140	RFC1 RFC14	1	1	0.78%
D6RAD2	Replication factor C subunit 1 (Fragment)	RFC1	1	1	6.82%
E0CX09	Replication factor C subunit 1	RFC1	1	1	6.92%
P37108	Signal recognition particle 14 kDa protein (SRI	SRP14	1	1	10.29%
H0YLA2	Signal recognition particle 14 kDa protein	SRP14	1	1	12.17%
P39023	60S ribosomal protein L3 (HIV-1 TAR RNA-binc	RPL3 OK/SV	1	1	2.98%
B3KS36	cDNA FLJ35376 fis, clone SKMUS2004044, highly similar to		1	1	3.39%
B5MCW2	60S ribosomal protein L3 (Fragment)	RPL3	1	1	4.41%
Q49AJ9	RPL3 protein (Ribosomal protein L3, isoform C	RPL3 hCG_1	1	1	4.78%
G5E9G0	60S ribosomal protein L3 (Ribosomal protein L	RPL3 ASC-1	1	1	3.42%
Q8TBW1	Putative uncharacterized protein (Fragment)		1	1	3.21%
Q9NY85	Ribosomal protein L3 (Fragment)	rpl3	1	1	3.50%
Q9BT63	RPL3 protein (Fragment)	RPL3	1	1	4.11%
P41182	B-cell lymphoma 6 protein (BCL-6) (B-cell lym	BCL6 BCL5	1	1	1.13%
A5PL18	B-cell CLL/lymphoma 6	BCL6	1	1	1.13%
B4E2L2	cDNA FLJ60220, highly similar to B-cell lymphoma 6 prote		1	1	1.18%
P47914	60S ribosomal protein L29 (Cell surface hepari	RPL29	1	1	9.43%
Q6IPI1	Ribosomal protein L29	RPL29	1	1	9.32%
Q5T1D1	Deleted.		1	1	9.55%
P49207	60S ribosomal protein L34	RPL34	1	1	7.69%
P55735	Protein SEC13 homolog (SEC13-like protein 1)	SEC13 D3S1	1	1	3.42%
Q53GB2	SEC13-like 1 isoform b variant (Fragment)		1	1	3.42%
E7ERC8	Protein SEC13 homolog (Fragment)	SEC13	1	1	12.79%
Q59FA6	SEC13-like 1 isoform b variant (Fragment)		1	1	3.63%
A8MWR8	cDNA FLJ59881, highly similar to SEC13-related protein		1	1	5.67%
A4UCS7	SEC13-like 1 isoform (Fragment)		1	1	6.21%
A8MXL6	Protein SEC13 homolog	SEC13	1	1	3.87%
P60174	Triosephosphate isomerase (TIM) (EC 5.3.1.1)	TPI1 TPI	1	1	4.20%
Q53HE2	Triosephosphate isomerase (EC 5.3.1.1) (Fragment)		1	1	4.82%
B4DUI5	Triosephosphate isomerase (EC 5.3.1.1)		1	1	5.63%
Q2QD09	Triosephosphate isomerase (EC 5.3.1.1) (Fragment)		1	1	4.82%
P61247	40S ribosomal protein S3a (v-fos transformati	RPS3A FTE1	1	1	3.41%
A8K4W0	40S ribosomal protein S3a	RPS3A	1	1	3.41%
D6RED7	40S ribosomal protein S3a (Fragment)	RPS3A	1	1	8.91%
D6RAT0	40S ribosomal protein S3a	RPS3A	1	1	3.96%
Q6NXR8	40S ribosomal protein S3a	RPS3A	1	1	3.41%
D6RG13	40S ribosomal protein S3a (Fragment)	RPS3A	1	1	4.04%
H0Y8L7	40S ribosomal protein S3a (Fragment)	RPS3A	1	1	4.74%
D6RGE0	40S ribosomal protein S3a (Fragment)	RPS3A	1	1	13.85%
F5H4F9	40S ribosomal protein S3a	RPS3A	1	1	5.14%
D6RAS7	40S ribosomal protein S3a (Fragment)	RPS3A	1	1	7.20%
E9PFI5	40S ribosomal protein S3a	RPS3A	1	1	4.39%
D6RB09	40S ribosomal protein S3a (Fragment)	RPS3A	1	1	4.64%
B7Z3M5	40S ribosomal protein S3a	RPS3A	1	1	5.14%

P62081	40S ribosomal protein S7	RPS7	1	1	4.64%
P62424	60S ribosomal protein L7a (PLA-X polypeptide	RPL7A SUR	1	1	4.89%
Q9BY74	Ribosomal protein L7a (Fragment)	RP-L7a	1	1	9.15%
Q5T8U2	60S ribosomal protein L7a (Ribosomal protein	RPL7A RP1:	1	1	8.61%
Q5T8U3	60S ribosomal protein L7a (Ribosomal protein	RPL7A RP1:	1	1	6.81%
P62750	60S ribosomal protein L23a	RPL23A	1	1	8.33%
B9EJE3	60S ribosomal protein L23A like (Uncharacteri	RPL23AL	1	1	8.33%
H7BY10	60S ribosomal protein L23a (Fragment)	RPL23A	1	1	8.23%
K7ERT8	DNA ligase 3 (Fragment)	LIG3	1	1	7.43%
A8MUS3	60S ribosomal protein L23a (Ribosomal protei	RPL23A hC(	1	1	6.70%
K7EMA7	60S ribosomal protein L23a	RPL23A	1	1	18.57%
K7EJV9	60S ribosomal protein L23a (Fragment)	RPL23A	1	1	7.65%
P62826	GTP-binding nuclear protein Ran (Androgen re	RAN ARA24	1	1	4.63%
B5MDF5	GTP-binding nuclear protein Ran (RAN, memb	RAN hCG_1	1	1	4.29%
J3KQE5	GTP-binding nuclear protein Ran (Fragment)	RAN	1	1	4.27%
B4DV51	GTP-binding nuclear protein Ran (cDNA FLJ52:	RAN	1	1	7.81%
F5H018	GTP-binding nuclear protein Ran (Fragment)	RAN	1	1	5.03%
P62906	60S ribosomal protein L10a (CSA-19) (Neural p	RPL10A NE	1	1	3.69%
Q1JQ76	Ribosomal protein (Fragment)	RPL10A	1	1	3.88%
Q00610	Clathrin heavy chain 1 (Clathrin heavy chain o	CLTC CLH17	1	1	0.66%
Q01844	RNA-binding protein EWS (EWS oncogene) (Ev	EWSR1 EW	1	1	1.37%
C9JGE3	Ewing sarcoma breakpoint region 1, isoform C	EWSR1 hC(	1	1	1.54%
B0QYK0	RNA-binding protein EWS	EWSR1	1	1	1.46%
H7BY36	RNA-binding protein EWS (Fragment)	EWSR1	1	1	2.92%
Q07065	Cytoskeleton-associated protein 4 (63-kDa cyt	CKAP4	1	1	1.83%
Q6NWZ1	CKAP4 protein (Fragment)	CKAP4	1	1	1.83%
Q8TB01	Similar to cytoskeleton-associated protein 4 (Fragment)		1	1	1.96%
B3KVX6	cDNA FLJ41699 fis, clone HCHON2004776, highly similar t		1	1	2.11%
Q07283	Trichohyalin	TCHH THH	1	1	0.67%
A2RRS3	TCHH protein	TCHH	1	1	3.76%
Q13243	Serine/arginine-rich splicing factor 5 (Delayed	SRSF5 HRS	1	1	3.31%
B4DJK0	Serine/arginine-rich-splicing factor 5 (cDNA FL	SRSF5	1	1	7.26%
F6T1J1	Serine/arginine-rich-splicing factor 4	SRSF4	1	1	5.70%
A8K588	cDNA FLJ76823, highly similar to Homo sapiens splicing fa		1	1	2.62%
B4DUA4	Serine/arginine-rich-splicing factor 5 (cDNA FL	SRSF5	1	1	6.52%
Q59EK7	CS0DF038YO05 variant (Fragment)		1	1	2.76%
Q59GY3	Arginine/serine-rich splicing factor 6 variant (Fragment)		1	1	3.23%
Q53F45	Splicing factor, arginine/serine-rich 4 variant (Fragment)		1	1	2.36%
Q59EF5	Splicing factor, arginine/serine-rich 4 variant (Fragment)		1	1	2.15%
B4DEM8	cDNA FLJ50118, highly similar to Splicing factor, arginine/		1	1	5.70%
A8K644	Splicing factor, arginine/serine-rich 4, isoform	SFRS4 hCG_	1	1	1.82%
Q08170	Serine/arginine-rich splicing factor 4 (Pre-mRN	SRSF4 SFRS	1	1	1.82%
Q13247	Serine/arginine-rich splicing factor 6 (Pre-mRN	SRSF6 SFRS	1	1	2.62%
Q13547	Histone deacetylase 1 (HD1) (EC 3.5.1.98)	HDAC1 RPD	1	1	2.49%
Q6IT96	Histone deacetylase (EC 3.5.1.98)	HDAC1 hC(	1	1	2.49%
B3KUJ5	Histone deacetylase (EC 3.5.1.98)		1	1	2.62%
Q92769	Histone deacetylase 2 (HD2) (EC 3.5.1.98)	HDAC2	1	1	2.46%
B3KRS5	Histone deacetylase (EC 3.5.1.98)	HDAC2 hC(	1	1	2.62%
F5GXM1	Histone deacetylase 1	HDAC1	1	1	4.15%
Q5TEE2	Histone deacetylase 1 (Fragment)	HDAC1	1	1	5.69%

J3KPW7	Histone deacetylase 2	HDAC2	1	1	2.06%
B5BU61	Histone deacetylase (EC 3.5.1.98)	HDAC1	1	1	2.49%
B4DRG0	cDNA FLJ51764, highly similar to Histone deacetylase 1		1	1	4.15%
B4DSK9	cDNA FLJ60860, highly similar to Histone deacetylase 1		1	1	5.06%
B7Z3S4	cDNA FLJ51981, highly similar to Histone deacetylase 1		1	1	6.90%
Q14103	Heterogeneous nuclear ribonucleoprotein D0	HNRNPD AU	1	1	3.94%
B4DTC3	Heterogeneous nuclear ribonucleoprotein D0	HNRNPD	1	1	4.62%
Q12771	P37 AUF1		1	1	4.90%
D6RAF8	Heterogeneous nuclear ribonucleoprotein D0	HNRNPD	1	1	6.33%
B4E0W4	cDNA FLJ61020, highly similar to Heterogeneous nuclear r		1	1	6.97%
D6RF44	Heterogeneous nuclear ribonucleoprotein D0	HNRNPD	1	1	12.50%
H0Y8G5	Heterogeneous nuclear ribonucleoprotein D0	HNRNPD	1	1	5.36%
F5H6R6	Heterogeneous nuclear ribonucleoprotein D0	HNRNPD	1	1	6.97%
H0YA96	Heterogeneous nuclear ribonucleoprotein D0	HNRNPD	1	1	6.67%
Q14166	Tubulin--tyrosine ligase-like protein 12	TTLL12 KIA	1	1	1.09%
Q9BR23	Putative uncharacterized protein (Fragment)		1	1	3.33%
Q14839	Chromodomain-helicase-DNA-binding protein	CHD4	1	1	0.42%
Q12873	Chromodomain-helicase-DNA-binding protein	CHD3	1	1	0.40%
Q8TDI0	Chromodomain-helicase-DNA-binding protein	CHD5 KIAA	1	1	0.41%
Q5TG85	Chromodomain-helicase-DNA-binding protein	CHD5	1	1	0.99%
K7EMY3	Chromodomain-helicase-DNA-binding protein	CHD5	1	1	0.75%
B3KRD4	cDNA FLJ34067 fis, clone FCBBF3001914, highly similar to		1	1	0.82%
B3KY63	cDNA FLJ16830 fis, clone UTERU3022536, highly similar to		1	1	0.42%
B4DLC6	cDNA FLJ59330, highly similar to Chromodomain helicase		1	1	0.82%
Q659F1	Putative uncharacterized protein DKFZp434K2	DKFZp434K	1	1	0.76%
Q659D0	Putative uncharacterized protein DKFZp434P2	DKFZp434P	1	1	0.75%
F5GWX5	Chromodomain-helicase-DNA-binding protein	CHD4	1	1	0.42%
Q3KNS1	Patched domain-containing protein 3 (Patche	PTCHD3 PT	1	1	1.30%
Q68DX3	FERM and PDZ domain-containing protein 2 (F	FRMPD2 PD	1	1	0.76%
B4E1N9	cDNA FLJ53430, highly similar to Homo sapiens	FERM and	1	1	1.19%
F8WCT2	FERM and PDZ domain-containing protein 2	FRMPD2	1	1	0.78%
Q6ZMW3	Echinoderm microtubule-associated protein-li	EML6 EML5	1	1	0.41%
Q7L2E3	Putative ATP-dependent RNA helicase DHX30	DHX30 DDX	1	1	0.67%
H7BXY3	Putative ATP-dependent RNA helicase DHX30	DHX30	1	1	0.69%
Q7Z403	Transmembrane channel-like protein 6 (Epide	TMC6 EVER	1	1	0.99%
K7ENC4	Transmembrane channel-like protein 6 (Fragn	TMC6	1	1	5.23%
B4E0Z0	cDNA FLJ59111, moderately similar to Transmembrane ch		1	1	6.56%
B3KTU5	Transmembrane channel-like protein		1	1	1.40%
K7ERH0	60S ribosomal protein L22 (Ribosomal protein	RPL22 hCG_	1	1	2.80%
K7ENM6	Transmembrane channel-like protein 6 (Fragn	TMC6	1	1	5.59%
Q86VX9	Vacuolar fusion protein MON1 homolog A	MON1A SA	1	1	1.26%
Q8IV63	Inactive serine/threonine-protein kinase VRK3	VRK3	1	1	2.32%
M0QYA8	Urokinase plasminogen activator surface rece	PLAUR	1	1	2.71%
B4DGW1	Inactive serine/threonine-protein kinase VRK3	VRK3	1	1	4.53%
M0R073	Urokinase plasminogen activator surface rece	PLAUR	1	1	3.04%
Q8IXQ6	Poly [ADP-ribose] polymerase 9 (PARP-9) (EC 2	PARP9 BAL	1	1	1.52%
B7Z5L6	cDNA FLJ54723, highly similar to Poly (ADP-ribose) polym		1	1	1.67%
Q8IYJ3	Synaptotagmin-like protein 1 (Exophilin-7) (Pr	SYTL1 SLP1	1	1	1.25%
Q8N880	cDNA FLJ39853 fis, clone SPLEN2014911		1	1	2.75%
A8KAH3	cDNA FLJ75170, highly similar to Homo sapiens synaptota		1	1	1.25%

Q8N999	Uncharacterized protein C12orf29	C12orf29	1	1	3.08%
Q8N9M5	Transmembrane protein 102 (Common beta-c	TMEM102	1	1	3.15%
Q8NAB2	Kelch repeat and BTB domain-containing prot	KBTBD3 BK	1	1	1.15%
G3V161	Kelch repeat and BTB (POZ) domain containin	KBTBD3 hC	1	1	1.31%
A8K1K0	cDNA FLJ76341, highly similar to Homo sapiens	kelch repe	1	1	1.14%
Q8NCA5	Protein FAM98A	FAM98A	1	1	2.89%
B4DT23	Protein FAM98A (cDNA FLJ52744, highly simil	FAM98A	1	1	4.64%
B4DY25	cDNA FLJ55866, highly similar to Protein FAM98A		1	1	4.30%
E9PH82	Protein FAM98A	FAM98A	1	1	4.81%
I6L9E8	Family with sequence similarity 98, member A	FAM98A	1	1	2.90%
Q8TDB6	E3 ubiquitin-protein ligase DTX3L (EC 6.3.2.-) (	DTX3L BBA	1	1	2.03%
Q8WXX0	Dynein heavy chain 7, axonemal (Axonemal b	DNAH7 KIA	1	1	0.15%
Q92784	Zinc finger protein DPF3 (BRG1-associated fac	DPF3 BAF4	1	1	1.32%
B7Z276	cDNA FLJ57274, highly similar to Zinc-finger protein Dpf3		1	1	1.36%
F8W7T1	Zinc finger protein DPF3	DPF3	1	1	1.21%
F5H575	Zinc finger protein DPF3	DPF3	1	1	1.36%
Q93008	Probable ubiquitin carboxyl-terminal hydrolas	USP9X DFF	1	1	0.43%
D3DWB6	Ubiquitin carboxyl-terminal hydrolase (EC 3.4.	USP9X hCG	1	1	0.46%
Q96I24	Far upstream element-binding protein 3 (FUSI	FUBP3 FBP	1	1	1.75%
Q96IX5	Up-regulated during skeletal muscle growth p	USMG5 DA	1	1	25.86%
Q96RR1	Twinkle protein, mitochondrial (EC 3.6.4.12) (I	PEO1 C10o	1	1	2.49%
E5KSY5	Mitochondrial twinkle protein		1	1	2.49%
Q9H6V3	C10orf2 protein (cDNA: FLJ21832 fis, clone HE	C10orf2	1	1	3.36%
Q99460	26S proteasome non-ATPase regulatory subur	PSMD1	1	1	1.05%
Q05CW6	PSMD1 protein (Fragment)	PSMD1	1	1	1.17%
Q05BX4	PSMD1 protein (Fragment)	PSMD1	1	1	1.21%
B2R6D0	cDNA, FLJ92896, highly similar to Homo sapiens	proteaso	1	1	1.05%
Q9BVJ7	Dual specificity protein phosphatase 23 (EC 3.	DUSP23 LD	1	1	8.00%
Q9C091	GREB1-like protein	GREB1L C1	1	1	0.36%
B4DDS9	cDNA FLJ53878, weakly similar to Homo sapiens	GREB1 p	1	1	0.72%
Q9H0U4	Ras-related protein Rab-1B	RAB1B	1	1	3.98%
Q92928	Putative Ras-related protein Rab-1C (hRab1c)	RAB1C	1	1	3.98%
Q6FIG4	RAB1B protein	RAB1B	1	1	3.98%
E9PLD0	Ras-related protein Rab-1B	RAB1B	1	1	4.73%
Q9NZB2	Constitutive coactivator of PPAR-gamma-like	FAM120A C	1	1	1.16%
Q9P219	Protein Daple (Coiled-coil domain-containing	CCDC88C D	1	1	0.30%
Q9Y4L1	Hypoxia up-regulated protein 1 (150 kDa oxyg	HYOU1 GRI	1	1	1.60%
E9PL22	Hypoxia up-regulated protein 1	HYOU1	1	1	1.71%
B3KXH0	cDNA FLJ45395 fis, clone BRHIP3027191, highly similar to		1	1	1.61%
B7Z766	cDNA FLJ54564, highly similar to 150 kDa oxygen-regulate		1	1	1.89%
B7Z2N4	cDNA FLJ56074, highly similar to 150 kDa oxygen-regulate		1	1	1.63%
B7Z602	cDNA FLJ59408, highly similar to 150 kDa oxygen-regulate		1	1	2.44%
A2N0U5	VH6DJ protein (Fragment)	VH6DJ	1	1	6.42%
A2N0T1	VH6DJ protein (Fragment)	VH6DJ	1	1	5.88%
A8K129	cDNA FLJ75032, highly similar to Homo sapiens	unc-84 hc	1	1	1.26%
B4E2A6	cDNA FLJ55508, highly similar to Sad1/unc-84-like protein		1	1	1.20%
B4DIU6	cDNA FLJ59542, highly similar to Sad1/unc-84-like protein		1	1	1.27%
J3KQE0	SUN domain-containing protein 2	SUN2	1	1	1.27%
Q6NT72	UNC84B protein (Fragment)	UNC84B	1	1	2.04%
Q9UH99	SUN domain-containing protein 2 (Protein un	SUN2 FRIG	1	1	1.26%

A8K3W4	cDNA FLJ75163, highly similar to Homo sapiens heteroger		1	1	1.98%
M0R3F1	ATP-dependent RNA helicase DDX3X (EC 3.6.4	DDX3X DBX	1	1	2.34%
A8K5K0	cDNA FLJ78309, highly similar to Homo sapiens heteroger		1	1	1.75%
A8K6U7	cDNA FLJ78252, highly similar to Homo sapiens heteroger		1	1	1.75%
Q9BUJ2	Heterogeneous nuclear ribonucleoprotein U-l	HNRNPUL1	1	1	1.75%
B7Z4B8	Heterogeneous nuclear ribonucleoprotein U-l	HNRNPUL1	1	1	1.96%
A8K6A2	cDNA FLJ77317, highly similar to Homo sapiens retinoblas		1	1	1.88%
H0YCT5	Histone-binding protein RBBP4 (Fragment)	RBBP4	1	1	4.44%
B4DRT0	Histone-binding protein RBBP4 (cDNA FLJ501	RBBP4	1	1	4.62%
H0YF10	Histone-binding protein RBBP4 (Fragment)	RBBP4	1	1	3.86%
H0YEU5	Histone-binding protein RBBP4 (Fragment)	RBBP4	1	1	4.79%
E9PC52	Histone-binding protein RBBP7	RBBP7	1	1	1.92%
Q5JNZ6	Histone-binding protein RBBP7 (Fragment)	RBBP7	1	1	9.41%
H0YDK2	Histone-binding protein RBBP4 (Fragment)	RBBP4	1	1	3.05%
Q5JP02	Histone-binding protein RBBP7 (Fragment)	RBBP7	1	1	4.57%
Q16576	Histone-binding protein RBBP7 (Histone acety	RBBP7 RBA	1	1	1.88%
Q6FHQ0	RBBP7 protein (Retinoblastoma binding prote	RBBP7 hCG	1	1	1.88%
Q09028	Histone-binding protein RBBP4 (Chromatin as	RBBP4 RBA	1	1	1.88%
A8K9U6	cDNA FLJ76121, highly similar to Homo sapiens zinc finge		1	1	1.44%
Q7Z2W4	Zinc finger CCCH-type antiviral protein 1 (ADP	ZC3HAV1 Z	1	1	1.44%
C9J6P4	Zinc finger CCCH-type antiviral protein 1	ZC3HAV1	1	1	1.27%
B1ALB6	Neutrophil cytosol factor 2 (Fragment)	NCF2	1	1	8.93%
Q9H9R6	cDNA FLJ12587 fis, clone NT2RM4001217, moderately sin		1	1	1.14%
Q9H0H3	Kelch-like protein 25 (Ectoderm-neural cortex	KLHL25 ENC	1	1	0.85%
B3KPR7	cDNA FLJ32101 fis, clone OCBBF2001176, highly similar to		1	1	0.90%
B1ALB7	Neutrophil cytosol factor 2 (Fragment)	NCF2	1	1	3.50%
Q59F14	Neutrophil cytosol factor 2 variant (Fragment)		1	1	0.84%
P19878	Neutrophil cytosol factor 2 (NCF-2) (67 kDa ne	NCF2 NOXA	1	1	0.95%
B1AUU8	Epidermal growth factor receptor substrate 1	EPS15	1	1	1.31%
Q7Z5V0	EPS15 protein		1	1	1.31%
B2R4C5	Lysozyme (EC 3.2.1.17) (Lysozyme (Renal amy	LYZ hCG_2	1	1	6.08%
F8VV32	Lysozyme C	LYZ	1	1	8.65%
P61626	Lysozyme C (EC 3.2.1.17) (1,4-beta-N-acetylm	LYZ LZM	1	1	6.08%
B2R4D8	60S ribosomal protein L27		1	1	6.62%
K7ELC7	60S ribosomal protein L27 (Fragment)	RPL27	1	1	6.25%
E4W6B6	RPL27/NME2 fusion protein (Fragment)	RPL27	1	1	7.14%
P61353	60S ribosomal protein L27	RPL27	1	1	6.62%
K7EQQ9	Transmembrane channel-like protein 6 (Fragn	TMC6	1	1	11.25%
B2R6F3	Splicing factor arginine/serine-rich 3 (Splicing	SFRS3 hCG_	1	1	8.54%
B4DEK2	cDNA FLJ59182, highly similar to Splicing factor, arginine/		1	1	8.48%
Q16629	Serine/arginine-rich splicing factor 7 (Splicing	SRSF7 SFRS	1	1	5.88%
B4E241	Serine/arginine-rich-splicing factor 3 (Splicing	SRSF3 SFRS	1	1	11.29%
C9JAB2	Serine/arginine-rich-splicing factor 7	SRSF7	1	1	5.96%
P84103	Serine/arginine-rich splicing factor 3 (Pre-mRN	SRSF3 SFRS	1	1	8.54%
B2RAN6	cDNA, FLJ95025, highly similar to Homo sapiens transcrip		1	1	2.22%
Q92754	Transcription factor AP-2 gamma (AP2-gamma	TFAP2C	1	1	2.22%
B4DWK3	Transcription factor AP-2 gamma (cDNA FLJ50	TFAP2C	1	1	3.56%
B2RE88	cDNA, FLJ96465, highly similar to Homo sapiens solute ca		1	1	3.32%
Q53HC3	Solute carrier family 25 member 3 isoform b variant (Frag		1	1	3.32%
Q00325	Phosphate carrier protein, mitochondrial (Pho	SLC25A3 PH	1	1	3.31%

Q8NCF7	cDNA FLJ90278 fis, clone NT2RP1000325, highly similar to		1	1	3.32%
F8VVM2	Phosphate carrier protein, mitochondrial	SLC25A3	1	1	3.70%
B3KX72	cDNA FLJ44920 fis, clone BRAMY3011501, highly similar t		1	1	0.93%
Q96BA7	HNRPU protein		1	1	0.97%
B4DLR3	cDNA FLJ54020, highly similar to Heterogeneous nuclear r		1	1	0.89%
Q00839	Heterogeneous nuclear ribonucleoprotein U (	HNRNPU H	1	1	0.85%
Q7Z4Q5	Heterogeneous nuclear ribonucleoprotein U (	HNRPU hC(	1	1	1.36%
B4DJW3	Renalase (cDNA FLJ55083, highly similar to Re	RNLS	1	1	3.02%
Q5VYX0	Renalase (EC 1.6.3.5) (Monoamine oxidase-C)	RNLS C10or	1	1	2.05%
B4DL14	ATP synthase subunit gamma	ATP5C1	1	1	4.00%
Q8TAS0	ATP synthase subunit gamma (Fragment)		1	1	3.44%
P36542	ATP synthase subunit gamma, mitochondrial (	ATP5C1 AT	1	1	3.36%
B4DRF4	Very-long-chain (3R)-3-hydroxyacyl-[acyl-carri	PTPLAD1	1	1	4.56%
H3BRL8	Very-long-chain (3R)-3-hydroxyacyl-[acyl-carri	PTPLAD1	1	1	5.71%
Q9P035	Very-long-chain (3R)-3-hydroxyacyl-CoA dehy	PTPLAD1 B	1	1	3.87%
H3BPZ1	Very-long-chain (3R)-3-hydroxyacyl-[acyl-carri	PTPLAD1	1	1	4.15%
H3BS72	Very-long-chain (3R)-3-hydroxyacyl-[acyl-carri	PTPLAD1	1	1	3.50%
H3BMZ1	Very-long-chain (3R)-3-hydroxyacyl-[acyl-carri	PTPLAD1	1	1	10.07%
B4DRX3	60S ribosomal protein L6		1	1	3.08%
Q8TBK5	60S ribosomal protein L6	RPL6	1	1	2.43%
Q9HBB3	60S ribosomal protein L6		1	1	2.42%
Q8N5Z7	60S ribosomal protein L6	RPL6	1	1	2.43%
Q02878	60S ribosomal protein L6 (Neoplasm-related p	RPL6 TXREB	1	1	2.43%
B2R4K7	60S ribosomal protein L6		1	1	2.43%
B4DUG4	cDNA FLJ51308		1	1	11.50%
K7ELG9	Protein LSM12 homolog	LSM12	1	1	5.65%
Q3MHD2	Protein LSM12 homolog	LSM12	1	1	6.67%
B4E0Q6	cDNA FLJ60209, highly similar to Transcriptional represso		1	1	4.20%
F5H7D9	Transcriptional repressor p66-alpha	GATAD2A	1	1	4.20%
B4DKZ7	Transcriptional repressor p66-alpha (cDNA FL.	GATAD2A	1	1	2.52%
B3KSZ4	cDNA FLJ37346 fis, clone BRAMY2021310, highly similar t		1	1	1.85%
Q86YP4	Transcriptional repressor p66-alpha (Hp66alpl	GATAD2A	1	1	1.74%
Q8WXI9	Transcriptional repressor p66-beta (GATA zinc	GATAD2B K	1	1	1.85%
B4E0V7	cDNA FLJ51709, highly similar to Myotubularin-related pr		1	1	1.13%
B4E119	cDNA FLJ58870, highly similar to Homo sapiens	NOL1/NO	1	1	8.64%
B5BTY4	ATP-dependent RNA helicase DDX3X	DDX3X	1	1	1.51%
B4E132	cDNA FLJ53122, highly similar to ATP-dependent RNA hel		1	1	2.46%
O15523	Heterogeneous nuclear ribonucleoprotein R (l	HNRNPR HI	1	1	1.52%
B4DXX7	Uncharacterized protein (cDNA FLJ50912, higl	DDX3Y	1	1	1.52%
B4DLU5	cDNA FLJ60675, highly similar to ATP-dependent RNA hel		1	1	1.88%
Q59GX6	DEAD/H (Asp-Glu-Ala-Asp/His) box polypeptide 3 variant		1	1	1.48%
Q5S4N1	Putative uncharacterized protein (Fragment)		1	1	2.76%
O00571	Myosin regulatory light chain 12B (MLC-2A) (N	MYL12B MI	1	1	1.51%
B7Z5Y3	cDNA FLJ52112, highly similar to C-myc promoter-binding		1	1	1.67%
A8K2M7	cDNA FLJ77039, highly similar to Homo sapiens	c-myc prc	1	1	1.19%
Q7Z401	C-myc promoter-binding protein (DENN doma	DENND4A I	1	1	0.59%
H3BTW5	C-myc promoter-binding protein (Fragment)	DENND4A	1	1	1.10%
Q05C90	DENND4A protein (Fragment)	DENND4A	1	1	1.18%
B7ZM99	MTHFD1L protein (Methylenetetrahydrofolate	MTHFD1L	1	1	0.92%
H0Y327	Monofunctional C1-tetrahydrofolate synthase	MTHFD1L	1	1	2.71%

Q5JYA5	Monofunctional C1-tetrahydrofolate synthase	MTHFD1L	1	1	7.50%
C9JAK5	ADP-ribosylation factor 4	ARF4	1	1	6.54%
P18085	ADP-ribosylation factor 4	ARF4 ARF2	1	1	5.56%
C9JPM4	ADP-ribosylation factor 4 (Fragment)	ARF4	1	1	7.87%
C9JU56	60S ribosomal protein L31 (Fragment)	RPL31	1	1	7.83%
H7C2W9	60S ribosomal protein L31 (Fragment)	RPL31	1	1	8.33%
B2R4C1	cDNA, FLJ92036, highly similar to Homo sapiens ribosoma		1	1	7.20%
B7Z4C8	60S ribosomal protein L31 (cDNA FLJ57527, hi	RPL31	1	1	6.92%
P62899	60S ribosomal protein L31	RPL31	1	1	7.20%
B8ZZK4	60S ribosomal protein L31	RPL31	1	1	11.39%
B7Z4E3	60S ribosomal protein L31 (cDNA FLJ58908, hi	RPL31	1	1	7.50%
C9JWC4	Holliday junction recognition protein (Fragme	HJURP	1	1	1.75%
Q8NCD3	Holliday junction recognition protein (14-3-3-a	HJURP FAK	1	1	1.34%
H7C3V8	Holliday junction recognition protein (Fragme	HJURP	1	1	3.73%
E1P5S2	RNA-binding protein 39 (RNA-binding region (	RBM39 RNI	1	1	4.56%
G3XAC6	RNA-binding protein 39 (RNA-binding region (	RBM39 RNI	1	1	4.02%
Q6N037	Putative uncharacterized protein DKFZp686A1	DKFZp686A	1	1	4.63%
Q14498	RNA-binding protein 39 (Hepatocellular carcin	RBM39 HC(	1	1	3.21%
B4DLM0	cDNA FLJ60296, highly similar to RNA-binding	region-cont	1	1	3.38%
B3KWX7	cDNA FLJ44170 fis, clone THYMU2035319, highly similar t		1	1	3.36%
Q68DD9	Putative uncharacterized protein DKFZp781C0	DKFZp781C	1	1	3.31%
B4DRA0	cDNA FLJ58459, highly similar to RNA-binding	region-cont	1	1	3.39%
Q6MZY7	Putative uncharacterized protein DKFZp686C1	DKFZp686C	1	1	4.56%
B7Z4L7	cDNA FLJ59035, highly similar to RNA-binding	region-cont	1	1	5.04%
B4E1M7	cDNA FLJ58465, highly similar to RNA-binding	region-cont	1	1	3.26%
H0Y4X3	RNA-binding protein 39 (Fragment)	RBM39	1	1	5.04%
E5RGB3	Tripartite motif-containing protein 35	TRIM35	1	1	2.63%
Q9UPQ4	Tripartite motif-containing protein 35 (Hemop	TRIM35 HL!	1	1	1.42%
E5RJH3	60S ribosomal protein L30	RPL30	1	1	10.71%
E5RI99	60S ribosomal protein L30 (Fragment)	RPL30	1	1	5.26%
O95757	Double-stranded RNA-binding protein Staufen	STAU1 STA	1	1	0.72%
Q53ZP9	Heat shock protein apg-1		1	1	0.72%
B4DZR0	cDNA FLJ55529, highly similar to Heat shock 70 kDa prote		1	1	0.69%
E9PDE8	Heat shock 70 kDa protein 4L	HSPA4L	1	1	0.74%
P62888	60S ribosomal protein L30	RPL30	1	1	5.22%
E7ES43	Heat shock 70 kDa protein 4L	HSPA4L	1	1	0.69%
B4DXT2	cDNA FLJ59635, highly similar to Heat shock 70 kDa prote		1	1	0.74%
E7EQV9	Ribosomal protein L15 (Fragment)	RPL15	1	1	5.17%
B4DLP4	Ribosomal protein L15		1	1	5.49%
P61313	60S ribosomal protein L15	RPL15 EC45	1	1	4.41%
E7EX53	Ribosomal protein L15 (Fragment)	RPL15	1	1	6.77%
E7ESJ3	Uncharacterized protein	ANKRD26	1	1	0.46%
A1L497	ANKRD26 protein	ANKRD26	1	1	0.46%
Q9UPS8	Ankyrin repeat domain-containing protein 26	ANKRD26 K	1	1	0.47%
A8K6L7	cDNA FLJ78668, highly similar to Homo sapiens deleted ir		1	1	0.92%
A8K119	cDNA FLJ76742, highly similar to Homo sapiens deleted ir		1	1	1.28%
Q96QB1	Rho GTPase-activating protein 7 (Deleted in liv	DLC1 ARHG	1	1	0.92%
E9PG32	Dynein heavy chain 12, axonemal (Fragment)	DNAH12	1	1	0.22%
Q6ZR08	Dynein heavy chain 12, axonemal (Axonemal I	DNAH12 DI	1	1	0.16%
E9PI69	Protein unc-93 homolog B1	UNC93B1	1	1	11.11%

E9PKU4	60S ribosomal protein L8 (Fragment)	RPL8	1	1	4.68%
E9PKZ0	60S ribosomal protein L8 (Fragment)	RPL8	1	1	5.37%
P62917	60S ribosomal protein L8	RPL8	1	1	4.28%
E9PP36	60S ribosomal protein L8	RPL8	1	1	7.43%
F2Z388	60S ribosomal protein L35	RPL35	1	1	10.42%
P42766	60S ribosomal protein L35	RPL35	1	1	8.13%
F4MHB8	Ubiquitously transcribed tetratricopeptide rep	UTY	1	1	3.29%
F4MHC9	Ubiquitously transcribed tetratricopeptide rep	UTY	1	1	1.13%
F4MH54	Ubiquitously transcribed tetratricopeptide rep	UTY	1	1	0.64%
F4MH35	Ubiquitously transcribed tetratricopeptide rep	UTY	1	1	0.48%
F5GZQ4	L-lactate dehydrogenase A chain (Fragment)	LDHA	1	1	20.83%
F5GXY2	L-lactate dehydrogenase A chain (Fragment)	LDHA	1	1	9.55%
F5GXH2	L-lactate dehydrogenase A chain (Fragment)	LDHA	1	1	13.16%
F5H6W8	L-lactate dehydrogenase A chain	LDHA	1	1	15.96%
F5GYU2	L-lactate dehydrogenase A chain (Fragment)	LDHA	1	1	10.42%
F5H5J4	L-lactate dehydrogenase A chain	LDHA	1	1	16.85%
F5H012	E3 ubiquitin-protein ligase TRIM21	TRIM21	1	1	1.89%
P19474	E3 ubiquitin-protein ligase TRIM21 (EC 6.3.2.-)	TRIM21 RN	1	1	1.89%
F5H1F7	Lysine-specific demethylase 5A	KDM5A	1	1	0.55%
P29375	Lysine-specific demethylase 5A (EC 1.14.11.-)	KDM5A JAF	1	1	0.53%
B4DVM9	cDNA FLJ50247, highly similar to Jumonji/ARID domain-co		1	1	0.86%
F8VRV5	Dynein light chain 1, cytoplasmic	DYNLL1	1	1	7.46%
F8VXI7	Dynein light chain 1, cytoplasmic (Fragment)	DYNLL1	1	1	11.90%
Q96FJ2	Dynein light chain 2, cytoplasmic (8 kDa dynei	DYNLL2 DL(	1	1	5.62%
Q6FGH9	DNCL1 protein (Dynein, light chain, LC8-type 1	DNCL1 DYN	1	1	5.62%
F8VXL2	Dynein light chain 1, cytoplasmic	DYNLL1	1	1	10.64%
A8K9V3	cDNA FLJ76469, highly similar to Homo sapiens tektin 1 (T		1	1	1.20%
F5GZX2	Tektin-1	TEKT1	1	1	1.84%
Q969V4	Tektin-1	TEKT1	1	1	1.20%
B4E2M7	cDNA FLJ52835, highly similar to Tektin-1		1	1	1.84%
P63167	Dynein light chain 1, cytoplasmic (8 kDa dynei	DYNLL1 DL(	1	1	5.62%
F8VWS0	60S acidic ribosomal protein P0	RPLP0	1	1	2.14%
F8VW21	60S acidic ribosomal protein P0 (Fragment)	RPLP0	1	1	2.46%
Q53HK9	Ribosomal protein P0 variant (Fragment)		1	1	1.89%
A8K4Z4	cDNA FLJ75549, highly similar to Homo sapiens ribosoma		1	1	1.89%
Q6NSF2	RPLP0 protein	RPLP0	1	1	2.36%
F8VS58	60S acidic ribosomal protein P0 (Fragment)	RPLP0	1	1	4.96%
F8VZS0	60S acidic ribosomal protein P0 (Fragment)	RPLP0	1	1	2.44%
G3V210	60S acidic ribosomal protein P0 (Ribosomal pr	RPLP0 hCG_	1	1	3.61%
F8VQY6	60S acidic ribosomal protein P0	RPLP0	1	1	4.23%
P05388	Myosin light chain 1/3, skeletal muscle isoforr	MYL1	1	1	1.89%
Q3B7A4	60S acidic ribosomal protein P0 (RPLP0 protei	RPLP0	1	1	2.35%
F8VRK7	60S acidic ribosomal protein P0	RPLP0	1	1	3.85%
F8VU65	60S acidic ribosomal protein P0 (Fragment)	RPLP0	1	1	2.43%
F8VYN4	60S acidic ribosomal protein P0	RPLP0	1	1	10.53%
Q53HW2	Ribosomal protein P0 variant (Fragment)		1	1	1.89%
B4E3D5	cDNA FLJ51469, highly similar to 60S acidic ribosomal pro		1	1	2.26%
Q8NHW5	60S acidic ribosomal protein P0-like	RPLP0P6	1	1	1.89%
H0UID3	Adaptor-related protein complex 2, beta 1 sul	AP2B1 hCG	1	1	1.37%
H0UID4	Adaptor-related protein complex 2, beta 1 sul	AP2B1 hCG	1	1	1.39%

P63010	AP-2 complex subunit beta (AP105B) (Adapter	AP2B1 ADT	1	1	1.39%
H0UID5	Adaptor-related protein complex 2, beta 1 sul	AP2B1 hCG	1	1	1.39%
A8K916	cDNA FLJ78481, highly similar to Homo sapiens	adaptor-r	1	1	1.37%
Q68DI0	Putative uncharacterized protein DKFZp781K0	DKFZp781K	1	1	1.37%
Q96EL6	Putative uncharacterized protein (Fragment)		1	1	2.34%
K7EJT8	AP-2 complex subunit beta	AP2B1	1	1	1.42%
B4DWG4	cDNA FLJ53223, highly similar to Adapter-relatedprotein (		1	1	1.42%
B4DIL5	cDNA FLJ54516, highly similar to Adapter-relatedprotein (		1	1	3.82%
B4E261	cDNA FLJ55646, highly similar to Adapter-relatedprotein (		1	1	1.89%
H0YC33	La-related protein 1 (Fragment)	LARP1	1	1	7.10%
E5RH50	La-related protein 1 (Fragment)	LARP1	1	1	2.13%
Q6PKG0	La-related protein 1 (La ribonucleoprotein dor	LARP1 KIAA	1	1	1.19%
H3BPX2	Obscurin	OBSCN	1	1	0.12%
Q5VST9	Obscurin (EC 2.7.11.1) (Obscurin-RhoGEF) (Ob	OBSCN KIA	1	1	0.14%
H3BSR6	Fractalkine	CX3CL1	1	1	1.74%
Q6I9S9	CX3CL1 protein	CX3CL1	1	1	1.76%
P78423	Fractalkine (C-X3-C motif chemokine 1) (CX3C	CX3CL1 FKN	1	1	1.76%
A0N0N7	Chemokine (C-X3-C motif) ligand 1 (cDNA, FLJ9	CX3CL1 hCG	1	1	1.76%
J3QRA1	Fractalkine	CX3CL1	1	1	1.95%
Q59GS2	Chemokine (C-X3-C motif) ligand 1 variant (Fragment)		1	1	1.84%
Q53FD8	Chemokine (C-X3-C motif) ligand 1 variant (Fragment)		1	1	4.12%
Q53H89	Chemokine (C-X3-C motif) ligand 1 variant (Fragment)		1	1	1.76%
H7C5K0	Helicase-like transcription factor (Fragment)	HLTF	1	1	2.12%
Q59GQ7	SWI/SNF-related matrix-associated actin-dependent regu		1	1	0.91%
Q14527	Helicase-like transcription factor (EC 3.6.4.-) (I	HLTF HIP11	1	1	0.89%
A8K5B6	cDNA FLJ76830, highly similar to Homo sapiens	SWI/SNF r	1	1	0.89%
I0B0K4	Truncated profilaggrin	FLG	1	1	0.63%
I0B0K7	Truncated profilaggrin	FLG	1	1	0.26%
I0B0K8	Truncated profilaggrin	FLG	1	1	0.17%
I0B0K6	Truncated profilaggrin	FLG	1	1	0.27%
I0B0K3	Truncated profilaggrin	FLG	1	1	0.85%
I0B0K5	Truncated profilaggrin	FLG	1	1	0.29%
P20930	Filaggrin	FLG	1	1	0.17%
I3L3P7	40S ribosomal protein S15a	RPS15A	1	1	9.00%
H3BT37	40S ribosomal protein S15a	RPS15A	1	1	18.00%
A8K7H3	cDNA FLJ77670, highly similar to Homo sapiens	ribosoma	1	1	6.92%
H3BV27	40S ribosomal protein S15a	RPS15A	1	1	18.37%
B2R4W8	HCG1994130, isoform CRA_a (cDNA FLJ30359	hCG_19941	1	1	6.92%
I3L246	40S ribosomal protein S15a	RPS15A	1	1	8.11%
P62244	40S ribosomal protein S15a	RPS15A OK	1	1	6.92%
H3BN98	Uncharacterized protein (Fragment)		1	1	3.80%
I3L303	40S ribosomal protein S15a	RPS15A	1	1	17.31%
I3L3Q7	Complement component 1 Q subcomponent-l	C1QBP	1	1	7.34%
A8K651	cDNA FLJ75700, highly similar to Homo sapiens	complem	1	1	4.61%
Q07021	Complement component 1 Q subcomponent-l	C1QBP GC1	1	1	4.61%
I3L3B0	Complement component 1 Q subcomponent-l	C1QBP hCG	1	1	7.30%
J3KPG5	Bromodomain adjacent to zinc finger domain	BAZ2A	1	1	0.32%
F8VU39	Bromodomain adjacent to zinc finger domain	BAZ2A	1	1	0.32%
H0YII3	Bromodomain adjacent to zinc finger domain	BAZ2A	1	1	2.93%
Q9UIF9	Bromodomain adjacent to zinc finger domain	BAZ2A KIAA	1	1	0.31%

J3QK86	Bromodomain adjacent to zinc finger domain	BAZ2A hCG	1	1	0.32%
J3QL05	Serine/arginine-rich-splicing factor 2 (Fragmer	SRSF2	1	1	6.15%
Q53FN0	Splicing factor, arginine/serine-rich 2 variant (Fragment)		1	1	3.62%
J3KP15	Serine/arginine-rich-splicing factor 2 (Fragmer	SRSF2	1	1	5.97%
Q9BRL6	Serine/arginine-rich splicing factor 8 (Pre-mRI	SRSF8 SFRS	1	1	2.84%
B3KX15	cDNA FLJ44468 fis, clone UTERU2026025, moderately sim		1	1	6.56%
B3KUY1	Splicing factor, arginine/serine-rich 2, isoform	SFRS2 hCG_	1	1	3.83%
Q01130	Serine/arginine-rich splicing factor 2 (Protein I	SRSF2 SFRS	1	1	3.62%
Q8N220	Splicing factor, arginine/serine-rich 2, isoform	SFRS2 hCG_	1	1	4.30%
B3KVY2	cDNA FLJ41718 fis, clone HLUNG2013097, highly similar t		1	1	4.10%
Q6NXQ0	SFRS2 protein	SFRS2	1	1	4.47%
B3KUF7	cDNA FLJ39750 fis, clone SMINT2017736, moderately sim		1	1	4.02%
Q8NAK9	cDNA FLJ35170 fis, clone PLACE6012942, highly similar to		1	1	3.98%
B4DN89	Serine/arginine-rich-splicing factor 2 (Splicing	SFRS2 SRSF	1	1	3.83%
K7EJT5	60S ribosomal protein L22 (Fragment)	RPL22	1	1	23.40%
K7EP65	DNA ligase 3 (Fragment)	LIG3	1	1	22.92%
K7EKS7	60S ribosomal protein L22	RPL22	1	1	20.75%
P35268	60S ribosomal protein L22 (EBER-associated p	RPL22	1	1	8.59%
K7EMH1	60S ribosomal protein L22 (Fragment)	RPL22	1	1	12.36%
K7ELC4	60S ribosomal protein L22 (Fragment)	RPL22	1	1	13.92%
K7ERI7	60S ribosomal protein L23a (Fragment)	RPL23A	1	1	11.58%
Q7Z4W8	Heparin-binding protein HBp15		1	1	8.59%
K7EKI0	Envoplakin	EVPL	1	1	0.29%
Q7RTP6	Protein-methionine sulfoxide oxidase MICAL3	MICAL3 KIA	1	1	0.30%
Q92817	Envoplakin (210 kDa cornified envelope precu	EVPL	1	1	0.30%
B7ZLH8	EVPL protein	EVPL	1	1	0.29%
J3QQT0	Protein-methionine sulfoxide oxidase MICAL3	MICAL3	1	1	2.22%
A8K0E1	cDNA FLJ78351		1	1	5.08%
Q8N4N8	Kinesin-like protein KIF2B	KIF2B	1	1	0.89%
B4DY48	cDNA FLJ53902, highly similar to Homo sapiens kinesin fa		1	1	1.07%
L0R809	Alternative protein EDARADD (EDAR-associate	EDARADD I	1	1	12.50%
M0QY97	Inactive serine/threonine-protein kinase VRK3	VRK3	1	1	0.66%
Q9UPT8	Zinc finger CCCH domain-containing protein 4	ZC3H4 C19	1	1	0.46%
M0QYR6	Urokinase plasminogen activator surface rece	PLAUR	1	1	3.98%
M0QYS6	40S ribosomal protein S11	RPS11	1	1	6.77%
M0R383	Heterogeneous nuclear ribonucleoprotein U-l	HNRNPUL1	1	1	5.84%
M0R1I2	Urokinase plasminogen activator surface rece	PLAUR	1	1	3.15%
M0R0Y4	Urokinase plasminogen activator surface rece	PLAUR	1	1	4.86%
Q03405	Urokinase plasminogen activator surface rece	PLAUR MO	1	1	2.69%
Q9UPI5	U-plasminogen activator receptor form 2-hum	PLAUR	1	1	3.59%
M0R0H5	Urokinase plasminogen activator surface rece	PLAUR	1	1	5.88%
M0R2E9	Urokinase plasminogen activator surface rece	PLAUR	1	1	5.92%
O60249	Heterogeneous nuclear ribonucleoprotein Q (	SYNCRIP HI	1	1	7.69%
Q8NI62	Ribosomal protein S2	OK/KNS-cl.	1	1	7.59%
Q8J014	Ribosomal protein S2	rps2	1	1	2.58%
Q8N5L9	Ribosomal protein S2	RPS2	1	1	2.05%
Q9BSW5	RPS2 protein (Fragment)	RPS2	1	1	6.19%
P15880	40S ribosomal protein S2 (40S ribosomal prot	RPS2 RPS4	1	1	2.05%
Q6ZNN8	CDNA FLJ27420 fis, clone WMC07143		1	1	3.95%
Q6IPX5	Ribosomal protein S2	RPS2	1	1	2.05%

A4D0Y7	Similar to 40S ribosomal protein S2	LOC392781	1	1	3.13%
H0YEN5	40S ribosomal protein S2 (Fragment)	RPS2	1	1	3.08%
Q3KQT6	Ribosomal protein S2	RPS2	1	1	2.05%
E9PQD7	40S ribosomal protein S2	RPS2	1	1	2.55%
Q05CK9	SYNCRIP protein (Fragment)	SYNCRIP	1	1	1.77%
Q6MZS5	Putative uncharacterized protein DKFZp686A1	DKFZp686A	1	1	1.31%
Q0VGD6	HNRPR protein (Fragment)	HNRPR	1	1	1.32%
B2R8Z8	cDNA, FLJ94136, highly similar to Homo sapiens synaptota		1	1	1.28%
B4DMB1	cDNA FLJ53358, highly similar to Heterogeneous nuclear r		1	1	1.34%
B2R7W4	cDNA, FLJ93632, highly similar to Homo sapiens heteroge		1	1	1.26%
O60506	Heterogeneous nuclear ribonucleoprotein C-li	HNRNPCL1	1	1	1.28%
B7Z645	Synaptotagmin binding, cytoplasmic RNA inte	SYNCRIP hC	1	1	1.72%
Q59GL1	Synaptotagmin binding, cytoplasmic RNA interacting prot		1	1	1.50%
O43390	Ribosomal protein S2 (Fragment)		1	1	1.26%
B4DT28	Heterogeneous nuclear ribonucleoprotein R (I	HNRNPR HI	1	1	1.62%
Q53G71	Calreticulin variant (Fragment)		1	1	2.22%
P27797	Calreticulin (CRP55) (Calregulin) (Endoplasmic	CALR CRTC	1	1	2.16%
B4DHR1	cDNA FLJ53009, highly similar to Calreticulin		1	1	4.25%
Q59GD4	Collagen, type V, alpha 3 preproprotein variant (Fragmen		1	1	0.42%
H0YH38	Probable phospholipid-transporting ATPase IK	ATP8B3	1	1	2.38%
P25940	Collagen alpha-3(V) chain	COL5A3	1	1	0.40%
Q5TEJ0	Replication protein A 32 kDa subunit (Fragme	RPA2	1	1	7.38%
B4DL94	cDNA FLJ51490, highly similar to Replication protein A 32		1	1	8.65%
P15927	Replication protein A 32 kDa subunit (RP-A p3	RPA2 REPA	1	1	3.33%
B4DQD9	cDNA FLJ61154, highly similar to Replication protein A 32		1	1	3.41%
B2R7E8	cDNA, FLJ93412, highly similar to Homo sapiens replicatic		1	1	3.33%
B4DUL2	cDNA FLJ57152, highly similar to Replication protein A 32		1	1	5.17%
Q65ZQ3	FBRNP	D10S102	1	1	3.72%
B4DDB6	Heterogeneous nuclear ribonucleoprotein A3,	HNRPA3 hC	1	1	2.81%
B4E3E6	cDNA FLJ58832, highly similar to Heterogeneous nuclear r		1	1	3.10%
P51991	Heterogeneous nuclear ribonucleoprotein A3	HNRNPA3 I	1	1	2.65%
Q6NSG7	High-mobility group nucleosome binding dom	HMGN1	1	1	10.00%
D3DWY4	HCG17955, isoform CRA_a	hCG_1795	1	1	10.00%
P05114	ADP/ATP translocase 2 (ADP,ATP carrier prote	SLC25A5 AI	1	1	10.00%
Q3B790	High-mobility group nucleosome binding dom	HMGN1	1	1	10.00%
A6NL93	Non-histone chromosomal protein HMG-14	HMGN1	1	1	11.11%
A6NEL0	Non-histone chromosomal protein HMG-14	HMGN1	1	1	8.62%
Q6NZ55	60S ribosomal protein L13	RPL13	1	1	3.79%
A8K4C8	60S ribosomal protein L13	RPL13 hCG_	1	1	3.79%
P26373	60S ribosomal protein L13 (Breast basic conse	RPL13 BBC	1	1	3.79%
J3QSB4	60S ribosomal protein L13 (Fragment)	RPL13	1	1	6.35%
Q86W20	Protease serine 1 (EC 3.4.21.4) (Fragment)	PRSS1	1	1	15.48%
Q7Z5F4	Protease serine 4 isoform B		1	1	4.98%
Q8N2U3	PRSS3 protein (Fragment)	PRSS3	1	1	5.18%
A1A508	PRSS3 protein	PRSS3	1	1	5.26%
B1AN99	Trypsin-3 (Fragment)	PRSS3	1	1	7.30%
P35030	Trypsin-3 (EC 3.4.21.4) (Brain trypsinogen) (M	PRSS3 PRSS	1	1	4.28%
Q8IWR8	Ribosomal protein L19 (Fragment)		1	1	5.20%
J3QR09	Ribosomal protein L19	RPL19	1	1	4.66%
Q53G49	Ribosomal protein L19 (Fragment)		1	1	4.59%

P84098	60S ribosomal protein L19	RPL19	1	1	4.59%
J3KTE4	Ribosomal protein L19	RPL19 hCG_	1	1	4.64%
Q8TA90	Similar to Elongation factor 2b (Fragment)		1	1	1.35%
E2DRY6	Enolase (EC 4.2.1.11)		1	1	2.07%
L0R849	Zinc finger CCCH domain-containing protein 4	ZC3H4	1	1	1.80%
Q9BV84	SEC16A protein (Fragment)	SEC16A	1	1	3.76%
A4QN18	SEC16A protein (Fragment)	SEC16A	1	1	0.97%
J3KNL6	Protein transport protein Sec16A	SEC16A	1	1	0.81%
A4QN19	SEC16A protein (Fragment)	SEC16A	1	1	1.10%
O15027	ATP-dependent RNA helicase DDX3Y (EC 3.6.4	DDX3Y DBY	1	1	0.87%
F1T0I1	Protein transport protein Sec16A	SEC16A	1	1	0.81%
Q9H2N7	HCLA-iso		1	1	1.72%
Q9HDB2	Putative uncharacterized protein CATX-3 (Frag	CATX-3	1	1	21.88%
S6C4R6	IgG L chain		1	1	3.80%
S6B2A1	IgG L chain		1	1	3.80%
P83593	Ig kappa chain V-IV region STH (Fragment)		1	1	6.42%
P06313	Ig kappa chain V-IV region B17		1	1	5.26%
P06314	ATP synthase subunit beta, mitochondrial (EC	ATP5B ATP	1	1	5.22%
P06312	Ig kappa chain V-IV region JI		1	1	5.79%
P01625	Keratin, type I cytoskeletal 14 (Cytokeratin-14	KRT14	1	1	6.14%

MW	PI
141519.2	7.53
228996.4	5.44
230774.4	5.46
75491.15	7.56
231213.5	5.52
232038.4	5.52
227867.9	5.52
62615.29	5.09
50669.62	9.39
70362.19	5.78
51560.92	5.09
66349.66	5.11
73679.65	5.87
73852.85	6.04
72400.2	5.7
69951.77	6.01
69719.99	5.84
68326.44	5.77
234233.9	5.64
227336.6	5.42
65625.31	9.69
65747.44	9.65
70670.07	9.52
63032.54	6.34
76120.21	8.7
76120.21	8.7
113082.5	8.99
113084.4	8.99
72332.07	5.07
56974.49	9.16
113372.2	8.7
112712.6	8.79
32004.07	8.88
69784.13	6.27
64239.73	9.28
64282.76	9.32
69842.23	6.23
17259.45	9.59
17259.45	9.59
17358.59	9.73
49670.28	4.78
52047.79	4.79
47766.13	4.7
46565.92	4.83
49670.28	4.78
51267.25	4.98
70782.06	9.31
67970.07	9.47

72359.8	9.38
28993.5	5.29
29096.29	9.74
29096.29	9.74
25566.54	9.69
29110.32	9.74
29082.27	9.74
25796.36	8.75
68137.42	6.92
22591.14	10.66
13161.26	10.45
16627.55	11.06
18563.87	10.81
18400.42	10.34
74222.44	5.95
140956.9	6.41
629094.1	5.8
82431.21	6.8
69554.9	7.62
82431.21	6.8
112905.7	9.17
106016.7	9.27
112905.7	9.17
20939.71	6.09
61696.41	9.33
82321.62	8.8
20456.67	4.62
19792.94	4.72
19778.95	4.71
19793.92	4.67
13997.33	10.32
18430.51	10.31
56200.33	6.63
48944.3	6.47
56101.2	6.5
24857.35	7.59
13508.51	10.58
13552.57	10.58
13162.96	9.99
49752.41	4.82
49839.49	4.83
49807.44	4.76
44601.78	4.84
49830.48	4.79
75022.25	9.7
22782.23	5.98
32574.66	4.64
29977.79	4.61
82703.57	5.55
64242.58	5.7

22349.61	11.02
21364.47	10.94
21864.98	11.03
21878.97	11.03
21864.98	11.03
21893.04	11.03
21893.04	11.03
40089.45	9.77
66407.58	5.11
37429.25	8.97
77514.87	8.84
17718.45	10.99
469083.6	6.75
465382.3	6.81
27744.25	10.37
25624.29	9.13
28328.85	10.09
47534.77	6.28
47500.76	6.28
18013.24	6.43
17971.14	7.73
18008.31	7.68
18012.3	7.68
29597.37	10.16
29578.33	10.11
27259.47	9.95
29597.37	10.16
48509.93	5.69
51027.65	5.19
51057.68	5.19
50975.66	5.39
47556.84	5.46
308772.9	9.11
316907.3	9.17
41904.84	10.44
35923.75	9.87
29374.12	10.23
29504.17	10.09
35122.98	10.01
50957.08	8.42
55804.35	6.44
39817.02	8.3
39419.55	8.3
39339.61	8.65
24823.4	6.81
24887.53	6.29
24700.19	6.41
24799.32	6.88
25023.66	7.59
10058.47	5.81

36910.7	7.8
92467.76	4.76
65952.54	5.08
92338.65	4.77
22579.88	10.91
29225.43	10.66
69475.67	6.02
65963.62	5.64
71013.21	5.75
43220.14	9.54
76613.47	4.6
68441.34	4.55
58553.77	4.57
65961.34	4.45
57220.64	9.22
358195.1	5.86
16272.53	10.07
17222.08	10.53
13015.29	11.01
12985.28	10.98
8217.79	10.1
22110.09	8.27
22200.26	8.58
87132.74	5.19
124343.9	6.18
55209.69	6.77
65820.82	7.27
61515.33	8.07
71173.63	7
66784.67	6.61
68965.12	6.99
63980.95	8.54
59153.3	8.78
51450.53	7.65
53600.22	9.14
58538.47	8.76
55943.51	8.66
55973.53	8.66
60562.56	9.17
67761.68	9.02
69147.29	9.06
69086.33	9.01
46011.85	11.36
47696.77	11.07
48995.28	11.02
37656.55	11.03
100645.4	5.56
47556.99	7.59
81066.53	8.22
80438.72	8.63

72574.86	8.82
80271.54	8.53
12918.06	11.92
16621.23	11.84
10333.96	11.82
15508.08	11.13
14052.44	11.34
15403.91	11.13
15387.84	11.27
55690.37	8.62
55674.33	8.62
54653.14	8.52
55083	5.45
60046.79	5.59
61053.94	5.7
60679.52	5.83
58570.18	5.82
32081.98	5.29
33611.58	5
22651.63	9.95
28915.87	8.39
31947.84	5.1
33669.62	4.95
32142	4.93
25256.31	9.83
32071.94	5.29
32029.09	5.48
32238.03	4.94
33570.49	4.95
31971.29	8.8
33280.3	5.11
88357.88	5.51
94464.97	5.82
99966.01	5.71
94622.15	5.87
9200.65	9.72
52736.67	9.66
62639.8	9.62
53927.96	9.65
43234.01	9.8
43266.08	9.8
56374.86	9.71
52624.61	9.73
58969.55	9.57
34861.49	9.53
53989.07	9.63
52768.73	9.66
38138.25	5.37
55307.87	5.25
48112.6	4.95

56559.22	5.26
18863	11.77
18731.93	11.82
18755.97	11.59
21728.28	11.73
21634.23	11.73
27486.2	7.14
27968.6	6
28067.73	6.19
28067.73	6.19
34910.38	6.28
34910.34	6.07
32014.53	6.63
34938.4	6.29
34165.96	9.3
19950.88	9.77
20252.15	9.64
20252.15	9.64
20094	9.64
14922.91	9.03
52163.66	5.42
31450.84	5.73
52129.6	5.36
52163.62	5.36
31510.94	5.73
52163.62	5.36
56423.24	6.52
22192.6	9.97
63181.55	9.46
22474.94	9.94
64745.56	9.47
54708.14	9.53
63191.58	9.46
56110.33	7.8
37275.71	8.47
57936.25	7.96
55925.94	7.2
49897.22	7.96
40163.12	8.16
53044.56	6.39
21144.87	4.97
21931.83	5.03
15603.49	4.73
28112.17	4.76
28082.08	4.76
10584.09	5.65
106649.1	9.26
48901.76	5.9
48882.71	5.87
10728.75	6.3

84870.34	6.18
75963.38	6.36
84872.31	6.18
57115.66	4.76
51187.09	4.82
55380.57	4.68
21252.37	4.47
51252.28	4.77
52501.53	4.76
51125.06	4.82
31432.75	4.51
31462.77	4.48
44551.72	6.1
20371.19	8.7
25711.52	6.24
25744.36	5.99
25674.44	6.91
25684.26	5.99
25561.07	5.6
25674.44	6.91
17389.88	6.08
27616.74	6.24
21134.13	8.38
27605.7	6.91
174383.1	8.82
33064.1	9.78
32851.84	9.71
32865.87	9.76
35293.5	9.83
17307.79	9.76
35021.46	9.91
32865.87	9.76
33080.1	9.78
35382.69	9.86
12537.52	11.07
6759.69	9.78
6472.46	10
10645.2	10.71
21396.8	10.18
19785.79	10.05
15959.33	10.53
20246.51	10.21
6504.49	10.35
12192.98	10.34
154725.6	5.14
166575.9	5.18
20170.26	10.1
271610.1	5.4
24636.11	6.7
20682.59	6.37

30357.58	6.24
59749.91	9.16
57546.41	9.31
29203.92	5.72
17818.38	9.48
21482.58	10.12
128253.1	9.38
14960.81	9.64
14761.71	9.72
14569.77	10.05
13058.06	9.62
46108.35	10.19
40144.26	10.3
31243	9.97
28581.21	10.17
40266.63	10.02
42752.46	10.01
39382.66	10.06
33737.04	10.07
78845.72	8.28
78792.68	8.18
75725.15	8.12
17751.86	11.66
17951.11	11.67
17367.36	11.24
13292.81	11.48
35540.12	5.22
35510.09	5.22
9630.83	6
33552.95	5.73
21415.71	6.03
19401.43	5.58
31594.96	5.47
30790.63	5.65
26712.2	6.9
22870.85	6.44
26942.47	8.21
29944.59	9.75
29954.63	9.75
11572.36	9.26
25886.78	9.46
29974.62	9.75
25607.68	9.68
22136.65	10.25
7285.28	9.43
20307.59	9.88
14445.62	9.86
23746.62	10.21
22572.38	9.78
20293.56	9.88

22126.56	10.09
29995.24	10.61
15636.27	10.06
16539.33	10.01
21544.51	11.02
17694.83	10.44
17507.29	10.21
17691.79	10.46
19639.93	10.54
21915.88	10.49
7923.19	9.22
19254.71	10.47
24422.81	7.01
26223.86	6.5
26815.72	9.62
14730.79	5.89
22534.81	8.87
24831.02	9.94
23535.56	10.08
191612.4	5.48
68477.49	9.37
61285.91	9.2
64928.66	9.42
32070.64	9.87
66021.66	5.63
67817.36	9.34
62059.66	5.51
58150.12	5.15
253922.2	5.73
44893.39	5.75
31263.45	11.59
14437.03	10.1
18268.95	5.15
39487.2	11.4
15809.69	10.12
37253.73	10.96
31864.94	10.95
44200.42	11.39
48084.21	11.79
18284.95	5.04
56707.71	11.52
56677.69	11.52
39586.34	11.42
55102.37	5.31
55102.37	5.31
51971.9	5.53
55363.6	5.59
51997.93	5.48
32728.27	4.78
24544.41	8.33

65537.21	6.54
55075.34	5.31
32704.29	4.75
27327.75	8.18
19747.18	6.22
38433.71	7.61
33611.76	8.62
31481.07	6.93
23076.22	5.03
22962.19	9.41
12640.42	8.61
29723.93	9.21
22961.21	9.47
23811	9.6
74402.76	5.33
23631.27	5.4
218002.1	5.62
226589.1	6.92
223047	5.82
92026.22	5.51
120867.5	5.89
113136.3	6.03
215282	5.65
110083.7	5.58
120891.5	5.89
121306.2	6.09
217101	5.59
86870.71	5.84
144280.1	6.29
93515.5	7.35
140669	6.24
217896.9	7.17
133936.6	8.99
130548.7	8.8
90044.23	8.98
17292.1	4.86
13607.89	4.79
63269.96	8.72
31679.22	9.25
16123.72	4.84
62130.7	5.9
52880.65	9.21
45157.63	9.3
27476.24	6.55
40523.72	9.23
96342.16	8.12
87549.46	8.4
61856.36	5.32
26695.05	5.07
61868.46	5.43

37489.59	6.6
54174.2	5.59
69394.17	5.22
60675.32	5.09
69852.74	5.18
55400.06	9.15
34086.39	10.02
36998.99	10.04
34430.9	8.58
55172.75	9.03
83553.41	8.31
461153.6	5.7
43083.78	6.01
41193.94	5.84
46444.25	6.93
41183.9	5.84
292276.5	5.52
271251.2	5.64
61639.7	8.6
6457.5	9.78
77153.26	9.13
77153.26	9.13
56989.5	9.32
105835.1	5.25
94729.11	6.86
91020.5	5.69
105837.1	5.25
16587.96	8.44
214351.9	6.18
108671	5.77
22170.9	5.55
22016.71	5.25
22197.93	5.55
18482.92	5.6
121886.3	9.07
228227.6	5.87
111334.1	5.16
104777.4	5.57
110439.9	5.15
94198.34	4.96
109704.3	5.87
72846.09	4.84
11963	5.16
12910.05	8.74
80366.94	6.35
83964	6.38
79082.93	6.47
79084.9	6.38
49423.03	5.5
80309.93	6.27

84820.78	8.99
71610.53	9.12
95647.4	7.52
95677.29	6.49
95737.39	6.49
86121.22	8.93
47837.57	4.93
20253.91	4.42
19484.07	4.44
23193.52	4.97
18992.09	5.3
46937.58	4.92
9525.15	3.76
30057.7	5.34
19523.54	4.96
47819.51	4.89
47819.51	4.89
47655.13	4.74
101455.3	8.68
101430.2	8.72
114084.3	8.86
6678.8	9.75
48955.65	6.47
65922.56	6.1
62350.4	5.82
16245.58	9.43
67587.64	5.9
59760.84	5.88
83653.44	4.56
83671.48	4.56
16536.86	9.38
11488.1	9.27
16536.86	9.38
15769.47	10.56
16358.86	10.59
14256.8	10.46
15797.53	10.56
9740.46	10.59
19329.38	11.64
18814.77	10.69
27366.23	11.83
14202.95	10.13
26927.75	11.86
19329.38	11.64
49144.13	7.72
49176.2	7.72
31009.48	9.14
39948.22	9.43
39928.23	9.43
40094.41	9.45

39932.18	9.43
36160.83	9.35
83084.08	8.95
79714.78	8.03
86859.6	6.07
90583.42	5.76
57662.65	9.42
25793.41	5.59
37846.89	6.06
27512.24	6.85
32246.77	9.13
32995.66	9.23
36430.37	8.88
29205.41	9.46
43159.02	9.04
40068.53	9.06
47117.61	8.99
16163.87	5.16
26072.76	10.67
32741.5	10.62
32890.68	10.59
32725.54	10.59
32727.51	10.59
32701.48	10.62
12508.06	8.38
24955.21	7.07
21700.51	7.62
28651.35	9.98
28623.33	9.94
46806.31	10.25
65258.03	9.77
68061.99	9.95
65260	9.73
61215.31	6.06
18195.07	6.04
73170.5	6.94
44841.14	6.43
73152.78	7.24
72930.63	8.4
58773.56	7.62
74577.99	7.72
40186.67	7.9
73242.57	6.73
74079.4	6.76
104834.6	8.6
209241.7	7.07
112606.9	8.18
105605.5	8.68
105887.9	8.32
36320.27	6.35

13203.25	9.33
17699.18	5.86
20510.54	6.6
14552.9	7.83
13335.52	10.79
12700.78	11
14494.75	10.54
15118.41	10.37
14462.69	10.54
8987.61	10.88
13955.18	10.66
64277.41	9.77
83538.2	9.4
29296.4	9.51
40540.74	5.94
48023.24	10.57
39817.91	6.12
59378.91	10.1
56332.72	10.4
56743.25	10.41
57443.69	10.28
56410.48	10.04
40526.72	5.94
36464	5.52
58404.87	10.24
36585.07	5.45
30593.76	5.96
56539.74	6.71
6262.62	10.67
12655.75	9.59
94511.45	5.63
94504.42	5.65
97560.66	5.56
91952.76	5.78
12783.92	9.65
97657.86	5.69
91938.73	5.78
20509.67	11.28
19338.35	11.93
24145.77	11.62
15722.15	11
198270.4	5.51
198210.3	5.51
196321.3	5.54
170579.5	5.97
122823.3	7.54
170589.5	5.98
267466.7	5.68
356937.8	5.85
13568.92	8.6

25593.52	11
22388.99	10.77
28024.32	11.04
16179.71	11.89
10644.63	10.3
14551.29	11.04
23851.09	6.53
68949.97	6.74
119747.3	8.07
159062.6	8.14
7875.25	4.5
17415.31	9.08
12482.44	5.78
10302.12	5.28
15845.38	6.58
9787.4	5.3
54081.04	6.06
54169.15	5.98
186257.7	6.41
192093.3	6.12
119429.6	5.95
7209.18	5.18
4771.37	4.6
10349.73	6.81
10365.77	6.89
5426.17	5.21
48224.51	6.08
31036.98	5.95
48282.54	5.98
30983.94	5.79
10365.77	6.89
30455.74	5.9
26900.98	9.05
34213.01	5.72
34239.09	5.72
27298.34	8.36
13828.37	9.83
26878.89	8.46
18270.23	9.65
15813.36	9.55
34273.11	5.72
27435.48	8.36
17441.4	9.57
27187.29	9.11
6785.86	9.74
34301.12	5.72
28538.24	4.69
34364.08	5.41
105474.4	5.25
104335.2	5.29

104551.4	5.22
104222	5.19
105700.6	5.16
105680.5	5.22
61082.01	4.84
101331.4	5.14
101303.3	5.14
38347.07	8.16
76242.86	5.08
20780.34	9.69
69902.18	6.19
123508.8	8.91
972993.3	5.7
868473.9	5.69
42761.77	6.08
42186.15	6.08
42202.2	6.08
42202.2	6.08
38055.23	5.48
40306.73	5.9
18355.81	8.33
42203.18	5.96
48033.06	8.92
111965.2	8.89
113927.4	8.82
113936.3	8.79
122549	9.3
294478.5	9.38
430402.3	9.28
277264.3	9.4
91099.41	9.36
259704.8	9.48
435164.4	9.24
11477.47	10.17
5750.04	11.05
14823.3	10.14
5671.93	11.05
14839.34	10.14
12303.27	9.18
14839.34	10.14
27167.94	9.6
6020.24	11.01
20005.6	4.1
31379.85	4.74
31361.88	4.74
20133.72	4.1
208073.7	6.22
210934.9	6.25
24041.6	10.28
211195.2	6.22

207813.4	6.25
15155.77	10.96
25502.14	11.86
15527.17	11.07
32287.16	11.72
13813.87	10.46
24189.65	11.94
25476.06	11.86
20450.19	9.88
22384.59	11.64
19442.98	9.81
21352.11	9.88
23194.63	11.83
24152.64	11.85
5082.75	9.4
5238.93	9.82
5733.52	10.16
14786.81	9.21
10421.77	9.65
9098.41	9.94
11168.4	6.35
14817.83	9.34
233827.8	6.9
224292.2	5.43
231601.3	6.56
233817.8	6.9
31093.88	9.48
14244.01	4.63
76252.93	8.89
63168.9	9.06
8711.89	9.58
95425.47	6.95
140255	5.87
25202.98	5.64
14863.41	5.64
16772.61	6.13
31637.74	6.65
20636.98	6.17
36977.63	6.19
27923.35	5.93
17140.06	5.43
17195.09	5.07
8710.82	7.99
8757.79	7.79
25604.91	9.88
31336.12	10.25
10540.95	9.29
31324.07	10.25
17002.34	9.97
31296.02	10.25

21068.43	9.98
21153.69	9.86
31384.17	10.25
25210.85	10.26
50649.86	6.27
68358.63	9.01
67860.06	9.01
69600.84	8.84
67006.9	8.42
70901.11	7.57
69601.78	8.68
52045.88	9.19
59606.09	8.82
70942.21	8.23
55717.49	9.26
46918.38	4.3
48140.96	4.29
24286.78	4.91
162769	6.25
33264.91	9.51
172118.6	6.44
13242.64	5.21
11134.31	5.84
29246.52	5.75
28542.72	5.9
29262.52	5.75
19062.24	5.45
29356.38	8.5
37028.63	8.46
34188.81	8.18
39594.48	9.1
10688.8	9.6
10884.2	9.99
10658.77	9.6
10674.73	9.52
9535.46	9.05
12434.56	9.4
24265.17	11.65
24261.18	11.65
24261.18	11.65
14692.13	12.12
9166.47	10.28
28198.66	6.15
27091.29	5.75
26486.8	6.87
19400.86	5.69
32528.49	7.46
20819.46	11.44
23134.28	11.48
23466.66	11.41

23465.71	11.48
23247.44	11.48
57499.72	6.51
36554.25	6.16
42341.02	5.64
54107.12	6.34
211072	5.31
251890.6	5.51
186491.4	5.25
233514.4	5.4
249479.1	5.54
39156.61	10.37
3393.78	6.74
20314.64	6.22
20372.68	5.37
12060.29	7.94
14632.35	6.15
14965.73	6.15
13379.89	5.09
12639.92	7.92

Supplementary Table 2. Real-time RT-PCR primers

Real-time RT-PCR primer	Sequence (5'-3')	Real-time RT-PCR primer	Sequence (5'-3')
β-actin-RT-F	CCAACCGCGAGAAGATGA	Trps1-RT-F	TCTACCAGAAGCTTCACTCG
β-actin-RT-R	CCAGAGGCGTACAGGGATAG	Trps1-RT-R	CTCTCTAACGGGCTTCCATT

Supplementary Table 3. ChIP-qPCR primers

Gene Symbols	Sense sequence (5'-3')	Anti-sense equence (5'-3')
CDC16	CATTGGTCTCTTCCCACTGA	CAAATGAAAGTCAAAGACAACCT
CCNA2	GTGCCCCAGATTTTAGACCT	GTCCGAAGGCTGACTCTAAG
CCNB1	AACAACACGGATACACCCCC	TCAGCGACTTAGATGACTTTGC
CDK1	ATGAGGTAGAAACAAAGCAC	AAGAAGGAAATGGAAAACT
Untr4	CTCCCTCCTGTGCTTCTCAG	AATGAACGTGTCTCCCAGAA
Untr12	TGGACCTTTACCTGCTTTATCA	AGCAAGGACTAGGATGACAGAA
